# The quantile domain volatility shock transmission between carbon emission trading system and European emerging stock markets: Practical implications for portfolio optimization

**DOI:** 10.1371/journal.pone.0349789

**Published:** 2026-06-08

**Authors:** Abdullah A. Aljughaiman, Mosab I. Tabash, Suzan Sameer Issa, Abdulateif A. Almulhim

**Affiliations:** 1 Finance Department, School of Business, King Faisal university, Al-Ahsa, Saudi Arabia; 2 College of Business, Al Ain University, Al Ain, United Arab Emirates; 3 Faculty of Administrative and Financial Sciences, University of Petra, Amman, Jordan; AAFT University of Media and Arts, INDIA

## Abstract

This article explores the asymmetrical volatility shock spillovers between European Carbon Emission Trading System (EU-ETS) and European emerging economies’ stock markets by using the Quantile-based Vector Auto Regression (QVAR), “Extended Joint” connectivity framework. The QVAR approach suggested that a volatility shock in the EU-ETS transmitted higher conditional volatility shocks of 19.37%, 17.86%, 18.9%, 19.49% and 19.76% towards the equity markets of Czech Republic, Slovakia, Greece, Hungary and Poland at the bullish quantiles (τ=0.95) as compared with bearish (τ=0.05) and moderate (τ=0.50) volatility conditions. The overall aggregated measure of the volatility shock spillovers between EU-ETS and equity markets is approximately 30.63% and 29.43% at bearish and moderate quantiles, respectively as compared with 88.01% at the bullish volatility conditions. The QVAR results also indicate that Greece, Poland, and Hungary (Slovakia) are structurally more (less) exposed to bearish and moderate EU-ETS volatility shocks. Therefore, Slovakia serves as a stabilizing asset within a multi-country portfolio, especially during bearish, bullish and moderate EU-ETS volatility conditions. Therefore, Slovakian equities remain least affected even in bullish EU-ETS volatility regime, functioning as an optimal low-beta hedge component and thereby help in reducing portfolio-wide shock amplification. Therefore, at the upper quantiles, where volatility transmission becomes uniformly elevated across all markets, fund managers take into account the volatility-convergence arbitrage, capitalizing on Slovakian equities persistent weaker sensitivity. However, stress testing frameworks should incorporate quantile-dependent country-specific volatility loadings, with higher coefficients for Greece, Poland, and Hungary across lower and moderate EU-ETS volatility shocks. In the terms of macro-prudential policy adjustments, equity market regulators and policy makers in Greece, Poland, and Hungary should strengthen transition-risk buffers during bearish and moderate ETS volatility shock diffusion. Finally, the DCC-GARCH-*t* copula findings suggested that portfolio managers can overweight Czech Republic and Slovakia equities during periods of rising EU-ETS volatility and use them as low-beta components in mixed carbon–equity portfolios.

## 1. Introduction

The firms’ resource capacity and broader macroeconomic expansions are often reflected in equity market performance [1]. The equity market performance is positioned as a barometer of broader economic vivacity [2]. Furthermore, the accelerating industrial growth and commercial activity within emerging economies typically amplifies energy utilization and demand. This has subsequently enlarged carbon output and elevates the financial burden associated with carbon allowance trading [3]. As a result, the upward shifts in the carbon prices tend to dampen equity returns by narrowing profit margins for enterprises with considerable carbon emissions. This is because the carbon intensive firms may require additional carbon allowances. Therefore, carbon intensive enterprises functioning with inadequate carbon allowances incur pronounced operational risks and escalating carbon abatement expenses, which can deteriorate their market performance as carbon prices climb [4]. This is due to the fact that environmental compliance frameworks played a contributory factor in reshaping the corporate financial outcomes by raising regulatory expenditures and the costs of integrating sophisticated emission-mitigation technologies [5]. On the other hand, sustainable green energy firms operating with excess carbon permits may capitalize on favorable carbon market cycles by selling vacant carbon allowances, thereby establishing the dynamic shock transmission linkages between EU-ETS and financial asset classes [6]. Therefore, rapid technological integration has also tightened linkages among emission-intensive industries, making them more vulnerable to cross-sector spillovers that may transmit volatility even to firms with relatively smaller carbon exposures [7,8]. The intensified economic momentum boosts energy usage and carbon emissions, augmenting the price of European Union Allowances [1]. This directional causality from carbon trading valuations towards the equity market returns familiarizes the shock transmission channel linking equity market volatility to European carbon price adjustments [9].

However, in the existing literature, studies mostly explore the symmetrical or linear association between carbon and financial asset classes from the developed economies by using the mean based connectivity approaches [1,9,10,11]. For example, in the context of Chinese economic development and expansion, a number of studies highlighted the symmetrical sock spillovers emanating within the China’s carbon trading system towards the equity returns through mean based connectedness approaches, most commonly based upon the granger causality and generalized vector autoregressive (VAR) specifications [12,13,7]. Whereas, in the context of Chinese higher and lower carbon intensive firms, Zhang and Xu [14] analyzed how disturbances propagate between commodity and financial asset classes by employing the linear connectedness approach by Diebold and Yilmaz [15]. However, evaluation of the asymmetrical and quantile-dependent transmission of volatility between the EU-ETS and developing European equity markets offers greater understanding of tail-risk episodes, such as systemic financial turmoil or abrupt regulatory transitions in carbon markets. A quantile VAR paradigm is well suited for this task, as it relaxes the assumption of normally distributed shocks and incorporates essential empirical properties of financial data, including skewness and heavy-tailed behavior [16–18]. These extreme dynamics typically receive limited attention within traditional mean-based VAR methodologies [19]. Nevertheless, existing literature has not yet examined heterogeneous nature of the volatility shock spillovers between MSCI-classified emerging European equity markets and the EU-ETS across varied quantiles such as bearish (τ=0.05), bullish (τ=0.95) and moderate (τ=0.50). This furthermore motivates to pose two important research questions: (1) Does the shock transmission mechanism between the European Union Carbon Emission Trading System (EU-ETS) and European emerging equity markets exhibit heterogeneity across different market volatility regimes, namely bearish, bullish, and moderate conditions? (2) To what extent is the hedging effectiveness (HE) against long-term volatility in the EU-ETS enhanced through short positions in European equity markets when employing optimal portfolio weight selection and hedge ratio strategies, as estimated via the DCC-GARCH-t copula model?

One of the motivation for exploring the asymmetric and quantile dependent volatility shock spillovers and dynamic portfolio hedging strategy between EU-ETS and European emerging equity markets is based upon the fact that the nonlinear and asymmetric connection with the EU-ETS is often impacted by factors including financial and economic downturns, sudden shifts in business cycle dynamics, and the complexity of economic systems, as noted by Kayani et al. (2024) [18] and Suleman et al. (2022) [20]. Financial crises, policy changes, international events like COVID-19, the geopolitical tension like Russia-Ukraine conflict, U.S. trade restrictions on China all have a constant impact on the stock market. These noteworthy occurrences therefore cause structural changes in equity returns [21], which result in non-independence and non-identical distribution patterns in the financial time series data [17]. However, Wu and Jiang [8] noted that although symmetrical Vector Auto-regression (VAR) models are useful for examining volatility spillovers between financial asset classes, they find it difficult to take into account nonlinear dynamics in complex relationships, especially those that are directionally dependent, asymmetrical, or dynamic [19]. The quantile-based spillover approach outperforms the mean-based approaches because it better captures the shock spillover mechanism in situations of bearish, bullish and moderate market volatility [22]. Therefore, traders, short-term speculators and shareholders might use quantile-specific models or tactics to lower risks and increase profits in order to solve this. Interestingly, analyzing this asymmetric connection makes it possible to determine if positive EU-ETS shocks have a stronger impact on stock returns than negative shocks [3]. The way investors adjust their expectations about corporate environmental performance is another fundamental cause of the asymmetric link between the EU-ETS and stock market volatility in Europe’s developing countries. In particular, investors have a tendency to respond disproportionately to little increases in company carbon intensity, which might result in the creation of ESG bubbles [23]. Therefore, this furthermore motivates us to explore whether the volatility shock spillovers from EU-ETS towards the European emerging economies equity markets is higher and more prevalent across bearish and moderate quantiles as compared with the bullish quantiles.

The motivational factor for exploring the dynamic hedging strategies through using both the hedge ratios and optimal portfolio weight selection approaches based upon DCC-GARCH-*t* copula framework between EU-ETS and European emerging economies’ equity returns is that the Visegrad V4 European developing nations are the sixth-largest economic bloc in the European Union, with a total GDP of 996 billion USD in 2019. Their GDP increased by an astounding 155% between 1991 and 2019. These nations spend more than EUR 14 billion a year, or 1.44% of their GDP, on research and development [24]. Additionally, the V4 countries have benefited immensely from EU investment, which has totaled EUR 340 billion. As the world’s largest carbon market, the European Union Emissions Trading System (EU-ETS) accounted for 87% of all emissions trading systems in 2023 with a valuation of €770 billion [25]. Furthermore, almost 12.5 billion metric tons of carbon credits were exchanged on international emissions markets, which is comparable to the amount seen in 2022 [26]. However, the whole market value increased due to skyrocketing prices in a significant European market. The cost of European Union Allowances reached a peak of €100 per metric ton of carbon dioxide in 2023. By 2023, emissions from electricity and industrial facilities throughout Europe have significantly decreased because to the EU-ETS, falling by almost 47% from 2005 levels [27]. In addition to the significance of the EU-ETS, stock markets are impacted by carbon emissions and reflect socioeconomic development [22].

Another motivational factor for incorporating the EU emerging economies (Czech Republic, Poland, Hungary, Greece and Slovakia) is that Czech Republic’s emissions intensity exceeds the EU average due to its highly energy-dependent industrial sector. The overall economic expansion of Czech Republic contributed 3.5% of Europe’s carbon emission. In addition, since 2005, the Czech Republic has been cutting emissions more slowly than the EU average [28]. With half of the fund transitioning towards the environmental projects, Poland has raised more than PLN 24 billion from auction revenues since 2013 to assist energy transition and climate change activities [29]. Poland’s stationary source emissions in 2022 totaled 184.1 million metric tons of carbon equivalent [30]. Whereas Hungary reduces its total carbon emissions by 32.5% between 2005 and 2023, which was somewhat more than the EU average of 30.5% within the same time frame and bounded for carbon neutrality by 2050 [31]. In 2019, Greece produced 86 metric tons of CO₂ equivalent. The Greek economy’s carbon intensity declined by 23% between 2005 and 2019 and this makes as major beneficiary from the EU-ETS. Additionally, the manufacturing and construction industries experienced the most significant emissions drop, with a 54% reduction [32]. In 2023, about 43.1% of Greek carbon emissions were sheltered by explicit EU-ETS [33]. The carbon emission of the Slovakia is higher than the EU average. While emissions from the energy sector decreased by 41% during the same time, accounting for 16% of total emissions in 2019, the industrial sector accounts for 37% of Slovakia’s carbon emissions [34]. Slovakia’s compliance and transition under EU policy directly contribute to the overall efficacy of the EU-ETS in lowering European emissions, as evidenced by the country’s significant emissions decrease of 41.7% under ETS [35]

Furthermore, the motivation for exploring the quantile domain shock transmission and dynamic hedging strategies between European emerging equity markets such as Poland, Greece, Hungary, Slovakia and Czech Republic and EU-ETS stems from their designation as the only European markets classified as “emerging” under the MSCI taxonomy. Existing literature has underscored their relevance within the broader regional financial architecture. For example, in the existing literature, Syriopoulos [36] explore the long-run co-movements exist between developed financial systems such as Germany and the principal Central European emerging economies including Poland, the Czech Republic, Slovakia, and Hungary and reported meaningful evidence of long-term interdependence. Likewise, Voronkova [37] examined the structural linkages between advanced markets in Western Europe and the United States and the major Central European emerging markets. The overall findings indicate that these emerging markets (Poland, Greece, Hungary, Slovakia and Czech Republic) demonstrate increasing integration with the global financial ecosystem, highlighting their susceptibility to externally transmitted shocks.

The **first** objective of this study is to investigate the transmission mechanism of shocks between the volatility of European emerging stock markets and the EU-ETS within a quantile framework, utilizing the “Extended Joint” QVAR model introduced by [38]. As a **second** objective, the study examines whether the hedging effectiveness (HE) against long-term volatility in the EU-ETS can be effectively reduced through short positioning in the MSCI European Visegrad emerging markets, i.e., Czech Republic, Hungary, Poland, Slovakia, and Greece [39]. For the said purpose, we utilize both the optimal portfolio weight selection and hedge ration methods, as proposed by [21,22], respectively, using the DCC-GARCH-t copula model.

*This study adds to the existing body of literature in several key aspects.*

***Firstly***, this is the first research article that shifts from the mean based symmetrical connectedness analysis between carbon–equity markets towards the asymmetrical and quantile-dependent nonlinear volatility shock transmission structure. Whereas, previous studies have explored the mechanism of shock transmission between the European carbon emission trading system and stock returns in the developed financial markets of Europe [1,11]. Moreover, prior studies have also investigated the linear or symmetric spillover shock transmission between the EU-ETS and stock returns [1,9,12,29–31]. Nevertheless, the symmetric connectedness approach is limited to analyzing uniform responses across the entire distribution, potentially overlooking critical tail dependencies that emerge during periods of market turbulence, such as extreme bearish or bullish quantiles, as well as during more stable periods represented by moderate quantiles. Therefore, the exploration of quantile dependent spillover of shocks extends existing studies relying upon contagion theory [32,33] but these studies only assume homogenous transmission of shocks between commodity and financial asset classes and negating the fact that EU-ETS may influence in a heterogeneity manner on equity market volatility during higher (stress) vs lower (calm) volatility regime. Prior studies exploring the carbon-finance nexus assume linear pass-through of carbon-price risk (34, 35). However, this research demonstrates that carbon-market shocks cause more persistent and higher contribution of volatility shocks at the higher quantiles (τ=0.95) as compared with the bearish (τ=0.05) and moderate (τ=0.50) and thereby affirming the possibility of non-homogenous volatility shock spillovers. This research article incorporates a framework in which the EU-ETS price shocks’ transmission dynamics are dependent on the quantile of the volatility distribution, linking carbon-stock markets’ shocks to real-time market volatility regimes. This aids in understanding the theoretical foundation that how EU carbon market regulatory uncertainty, free-allocation reforms, or EU market stability carbon reserve adjustments influence equity volatility differently in bearish vs. bullish volatility conditions.

***Secondly***, this research also highlights whether hedge-ratio methods or optimum portfolio-weighting strategies provide better hedging efficacy against long-term investment risk in the EU-ETS through short-term investment positioning in European developing equities markets. Furthermore, this research expands on previous work on portfolio diversification and risk-transmission studies on carbon and developed economies’ sustainable stocks [1] as well as commodities and sustainable financial asset classes [38,39] by analyzing the appropriate strategical approach for hedging effectiveness of EU emerging-market stocks against the long-term EU-ETS volatility. The identification that whether hedge ratio or optimal portfolio weight selection approach is suitable for long-term carbon risk hedging provides more robust protection against EU-ETS volatility, offering actionable guidance for traders, speculators and carbon-exposed firms. Furthermore, with the aid of DCC-GARCH-t copula approach, this research can better estimate the dynamic and tail-dependent co-movements between conventional EU emerging stocks and EU-ETS that conventional linear or Gaussian models fail to detect.

***Thirdly***, this research article also explores the time-varying asymmetric and nonlinear volatility transmission patterns between EU-ETS and EU emerging equity markets during the notable events like COVID-19, geopolitical uncertainty risk like Russian-Ukraine war and financial uncertainty events during the post COVID-19 era like SVB implosion. This approach also uncovers whether volatility shocks are amplified during market stress (bullish quantiles), persist in moderate conditions (bearish quantiles), or continue through lower volatility dynamics (bearish quantiles) that are obscured in average-based connectivity models. Therefore, on the methodological front, this study also integrates the Quantile Vector Auto-regression (QVAR) connectedness framework by Ando et al. [40] with the “Extended Joint” connectedness framework by Balcilar et al. [41] and augments the conventional mean-based connectedness approach of Diebold and Yilmaz [15]. The QVAR based “Extended Joint” connectivity approach can uncover the volatility shock spillovers not on across different volatility regimes but also in the presence of structural breaks, non-normality in data and leptokurtic data distribution [21]. This incorporation of advanced normalization techniques resolves interpretability challenges, ensuring that connectedness measures are robust across quantiles [38]. Together, QVAR approach provides a richer understanding of EU-ETS–equity market linkages and identifies when and under which market conditions carbon-market shocks are most influential, offering valuable insights for short-term speculators, traders and portfolio optimizers.

The QVAR findings show that the total volatility connectedness indices (TCI) between EU-ETS and EU emerging stock markets is more persistent and higher in magnitude at the higher quantiles as compared with the median and lower quantiles. For instance, at the higher quantiles, the aggregated measure of forecast error variances due to the overall spillover of volatility shocks within the entire QVAR system is 88.01% as compared with the median (29.43%) and bearish quantile (30.63%). Similarly, a volatility shock in the EU-ETS also causes the higher contributions of shocks of 95.37% at the higher quantile (τ=0.95) as compared with the 24.55% and 17.65% at the lower (τ=0.05) and median (τ=0.50) quantiles, respectively. This shows the heterogeneous volatility shock transmission mechanism between EU-ETS and EU emerging equity markets across bearish, bullish and moderate volatility regimes.

The QVAR findings also shows that a volatility shock in the EU-ETS lead towards the highest contributions of shocks of 5.27%, 6.32% and 5.66% towards the conditional volatility of Greece, Hungary and Poland at lower quantile and 5.02%, 4.47% and 3.68% at the median quantile. However, the EU-ETS transmitted higher volatility shocks towards these economies’ conditional volatility at the higher quantile as compared with the median and lower quantiles. For instance, at the higher quantile, EU-ETS transmitted the higher contributions of shocks of 19.37%, 18.9%, 19.49% and 19.76% towards the conditional volatility of Czech Republic, Greece, Hungary and Poland, respectively. The QVAR findings also suggested that Slovakian equity market remains the least susceptible to the EU-ETS volatility shocks across bearish, bullish and moderate volatility regime. These findings propose specific practical implications for fund managers as the results highlight the need to consider stress-testing EU emerging economies’ equity market portfolios under quantile-specific carbon-price scenarios, instead of emplacing mean-based correlations. Furthermore, the firms relying upon the carbon intensive energy resources and actively participate in ETS in Central and Eastern Europe should prepare for capital-market unpredictability during ETS upward price shifts, as bullish-quantile spillovers signal potential volatility shocks in Czech Republic, Greece, Hungary and Poland and thereby increases the balance-sheet risk. At all the quantiles, the regulators can also use these findings for the identification of the susceptible stock markets, particularly Greece, Hungary, and Poland, and design targeted carbon-policy communication strategies that reduce uncertainty during periods of ETS higher volatility regime. However, Slovakian equity markets’ conditional volatility received the least volatility shocks from EU-ETS volatility across all quantiles, the Slovakia’s equities may serve as the low-beta defensive asset in carbon-linked portfolios, specifically for portfolio managers seeking constancy profits during high ETS volatility. Additionally, policy makers should also put emphasis on the market-stability mechanisms and higher liquidity provision in the ETS during bullish volatility regime due to its higher volatility shock diffusion at higher quantiles towards the EU emerging equities.

The findings based upon the DCC-GARCH-t copula approach suggested that using a bivariate optimal portfolio weight selection approach by Kroner and Ng [42] is more efficient in minimizing risk than the hedge ratio strategy. This research significantly contributes by demonstrating that, to reduce risks associated with EU-ETS volatility, creating an optimal portfolio that includes the European emerging equity markets of the Czech Republic and Slovakia delivers the highest hedging effectiveness, achieving 85% and 90%, respectively, surpassing other European emerging stock markets. Furthermore, the optimal portfolio weights for EU-ETS and the stock returns of the Czech Republic and Slovakia are 0.12 and 0.16, respectively. This implies that higher allocations of 0.88 and 0.84 should be directed toward these equity markets to effectively reduce risk against EU-ETS volatility. The policymakers and institutional investors within emerging European economies should recognize the remarkable hedging capabilities demonstrated by the Czech and Slovakian equity markets. Additionally, the relative resilience of the Czech and Slovakian equity markets to fluctuations in carbon prices positions them as valuable assets for stabilizing investment portfolios during periods of uncertainty arising from carbon trading policies.

The structure of this article is as follows: Section 2 reviews the current literature on the asymmetrical connection between the EU-ETS and stock markets. Section 3 describes the data used in the analysis, while section 4 explains the methodological approach. Section 5 presents the empirical results and their practical significance, and section 6 offers the conclusions.

## 2. Literature review and economic rationality

One of the theoretical justification for the asymmetric quantile domain interactions between European carbon emission trading system and stock returns is that EU-ETS price fluctuations directly affect the cost structure and profitability expectations of carbon-intensive sectors [1]. This implies that stock markets with greater exposure to carbon intensive energy resources and non-internalization of sustainable mechanical processes of green energy utilization may respond more strongly to carbon-price uncertainty Wen at al. [3]. According to the cost-of-capital and investment-adjustment literature, large volatility shocks in carbon prices increase discount-rate uncertainty, firms’ profitability and earnings risk. Thereby, carbon intensive firms may respond more strongly to higher volatility in the carbon prices in an asymmetrical fashion as compared with the lower volatility [2]. Wen et al. [2] also shows that both the decrease and increase in carbon trading system lead towards the disproportionate and heterogeneous influence on the Chinese financial system. Furthermore, building upon the cross-market contagion literature [43,44], the extreme movements in one market lead to disproportionate co-movements in others due to herding, common investors, or liquidity shocks [45]. Therefore, there may be the possibility that shocks transmitted from EU-ETS are tail dependent and higher intensity of volatility shock spillovers emerges at bullish quantiles. This is due to the fact that extreme equity volatility is much higher during extreme carbon-market turbulence. For instance, Duan et al. [46] also classified that shock spillovers between carbon and financial asset classes are more intensified at varied quantiles. Thereby, the findings suggest the quantile dependent shock spillover mechanism between both the markets and this shock spillover mechanism is furthermore intensified in the presence of economic disruptions. Furthermore, few other studies have also suggested that structural breaks within the time series data and the possibility of the presence of outliers within the financial time series observation [5,16,17,24] during the specific financial recession regime like global economic crisis of 2008, geopolitical uncertainty of Russian-Ukraine war and COVID-19 may also justifies the utilization of quantile dependent shock spillovers between EU-ETS and European emerging stock markets. Therefore, we can infer that there is asymmetrical volatility contagion between EU-ETS and EU emerging stock markets. This furthermore justifies exploring whether the volatility shock spillovers from EU-ETS towards the EU emerging stock market conditional volatility is higher in magnitude and persistent at bullish quantiles as compared with the bearish and moderate quantiles.

One of the theoretical rationality for the higher volatility shock transmission from EU-ETS towards the EU emerging economies stocks is that in the times of growing uncertainty and volatility, liquidity in stock markets tends to disappear [47]. Furthermore, the emerging markets could see greater liquidity declines during periods of financial concern due to their comparatively lower sophistication and depth, virtually disappearance of market makers, and increased vulnerability to international stock mare integration [19]. Therefore, building upon this fact, liquidity diminished in the wake of escalating volatility periods and this lead towards the larger price impacts. Emerging markets, being less liquid, exhibit stronger sensitivity during higher uncertainty and volatility regimes [47]. Furthermore, another justification for the quantile domain interactions between EU-ETS and EU emerging member economies stock market is that quantile-based dependence models as presented by Koenker and Bassett [48] and Koenker and Xiao [49] further elaborated that financial time series display fat tails, asymmetric dependence, and nonlinear shock transmission [16]. In the wake of extreme uncertainties within the financial system like COVID-19 and geopolitical uncertainty like Russian-Ukraine war events, the financial time series data exhibit leptokurtic distribution and thereby rendering the mean based models ineffective for shock transmission. Therefore, during the bullish volatility regime, reduced liquidity causes higher volatility shock transmission effects of EU-ETS shocks on the emerging equities and this shock transmission effect become disproportionately large, explaining why QVAR models find stronger spillovers under high-volatility conditions. Therefore, we can infer that overall volatility shock transmission from EU-ETS towards the EU emerging stock market volatility is higher in the at the bullish quantile (τ=0.95) as compared with the bearish (τ=0.05) and moderate quantile (τ=0.50). As, [16] also confirms that financial asset classes receive higher shocks at higher quantiles as compared with the bearish and bullish during the economic uncertainty periods. Whereas, [50] highlight that overall shock transmission between financial asset classes differ across bearish and bullish market periods. Therefore, we can infer the heightened volatility shock transmission from EU-ETS towards the EU emerging stock market’s conditional volatility across different quantiles.

**H1:** Volatility shocks from the EU-ETS exert a significantly stronger and heterogeneous impact on the conditional volatility of EU emerging stock markets across different quantiles, with the intensity of transmission varying under distinct market conditions.

The prominent theoretical rational regarding the volatility shock transmission from EU-ETS towards the EU emerging stock markets is embedded in the theoretical approach provided by Ren et al. (2023). For instance, as merging economies provide an ideal environment for examining the impacts of carbon price volatility for two main reasons. For instance, developing economies relies on carbon trading to meet emission targets, making carbon price uncertainty a significant but underexplored risk. Second, developing stock market, characterized by high information asymmetry [5,23,46,47], thereby providing a valuable context for analyzing how carbon price uncertainty influences stock volatility in an asymmetrical fashion. Ren et al. [51] highlight two other ways in which carbon market returns influences stock price risk in emerging markets: management’s tendency to withhold negative information and the creation of varying investor expectations. Initially, heightened uncertainty in carbon prices jeopardies investments, uncertainties about future cash flows and financing limitations, leading management to hide unfavorable news (57).Whereas, firms with higher cash flow uncertainty are cautious in R&D investment and financial friction intensifies the negative impact of cash flow uncertainty on innovation [52] and thereby adversely affecting the firm’s profitability and stock risk [53]. Moreover, high carbon price volatility affects investment propensities, with detailed financial analysis and real options playing a significant role [54]. Second, growing investor attention to environmental issues and economic uncertainty heightens sensitivity to external information, causing divergent opinions among investors. Hidden bad news eventually surfaces, leading to overvalued stock price crashes [51]. Therefore, we can infer the heightened volatility shock transmission from EU-ETS towards the EU emerging stock market’s conditional volatility across different quantiles.

The economic rationality that how short investment positioning within the European emerging equity market returns for long-term EU-ETS volatility hedging can be explained with the aid of theoretical approaches embedded within the framework of modern portfolio and Arbitrage pricing theoretical assertions. For instance, building upon the theoretical assertion of Modern Portfolio Theory (Markowitz, 1952), the exhibition of negative connectedness or correlation between two asset classes (carbon and equity market returns) implies the higher hedging effectiveness. Furthermore, the appreciating carbon emission trading system prices may have caused elevated carbon related abatement and compliance costs for carbon-intensive firms. This may have caused lower valuation for the carbon intensive firms within the EU emerging financial system. Therefore, because of inverse association between carbon and emerging stock markets of EU, we can infer that a short investment positioning within the European Union (EU) emerging economies’ equity market returns may lead towards higher compensating gains during carbon market bullish tendency. This has caused higher variance reduction in a diversified portfolio. However, in the existing literature, Oestreich and Tsiakas [55] only investigated the dependence of developed economies’ financial market on the European carbon emission trading system and found that firms receiving free carbon permits outperforms firms that didn’t receive these free carbon allowances. Tabash et al. [5] also investigated the existing of inverse association between carbon and stock market returns and found that increase in European carbon trading market decreases the equity market returns of developed European financial markets (Spanish and Belgian) and this effect is more pronounced in the long-term and across higher quantiles. Whereas, in the context of Chinese economy, Liu et al. [56] also highlighted the inverse association between carbon and stock market returns and found that carbon market return’ fluctuations causes an adverse effect on the Chinese industrial equities. Ouyang et al. [57] highlighted the role of Chinese carbon trading system for predicting the equity market returns and findings emphasized that incorporating carbon trading system price fluctuations as a forecasting variable for stock returns may improve the forecasting precision and predictive accuracy. Furthermore, gaining insights from the arbitrage pricing theory, fluctuations within the carbon and stock returns are affected by joint systematic risk factors like energy related volatility [22,35,46], climate and regulatory shocks [see 57 and 58] and global geopolitical risk and economic uncertainty factors. Therefore, appreciative carbon trading system prices and carbon related abatement costs typically translate into negative equity returns for emission-intensive firms [1,3,5], implying that short equity positions can neutralize shared risk factor exposures. Therefore, we can infer that a short investment positioning within one of the European emerging stock returns causes higher risk mitigation and higher hedging effectiveness against long-term volatility within the EU-ETS.

**H2:** Short investment positioning in European emerging equity markets significantly enhances hedging effectiveness against long-term volatility in the EU Emissions Trading System (EU-ETS).

For instance, building upon the portfolio hedging framework, adopting a short investment positioning within the asset class (equity market returns) may serve as long-term risk hedging and offsetting the anticipated adverse price fluctuations within another asset class (carbon trading system) [5,11,49]. This may lead towards the reduction in overall portfolio systematic risk. Furthermore, another justification for the higher hedging effectives of short investment positioning within EU-ETS against carbon trading system’s long-term volatility is due to the market imperfections and behavioral inefficiencies. For instance, there may be possibility that carbon abatement costs may not be fully or instantaneously priced into equity markets and equity market returns may respond positively to elevated carbon related costs [55]. Such inefficiencies enhance the profitability and hedging potential of short positions.

In the existing literature, studies mostly take into account the symmetrical shock spillover methodologies such as generalized VAR and Time Varying Parameter VAR (TVP-VAR) based connectivity approaches in order to account for the shock received by the financial asset classes from the carbon trading system. For instance, in the European economic context, Ji et al. [58] analyzed the symmetrical shock spillovers between carbon and energy sectoral stock returns by using the linear VAR based network analysis. Aslan and Posh [10] also explored which European sectoral stock transmitted higher error variance transmission towards the carbon trading system by relying upon the mean based connectivity approach formulized by Diebold and Yilmaz [15]. Whereas, Qiu et al. [59] explore the connectivity between sustainable energy firms and carbon trading market by using the TVP-VAR approach. In a similar fashion, Suleman et al. [1] explore the sustainable stocks and carbon trading system of European financial system by using the mean based connectivity approach of Diebold and Yilmaz [15]. Following the similar trend, Zhang and Xu [14] also explore the shock spillover mechanism between Chinese carbon trading prices and sustainable and conventional firms. More recently, Yadav et al. [60] explored the mean based connectedness mechanism between energy related commodities and carbon trading system and found that energy related commodity are the main shock transmitters within the generalized VAR based system of shock spillovers. However, no efforts are made to explore the asymmetric quantile dependent volatility shock transmission mechanism between EU-ETS and European emerging stock markets. This means that prior studies have not explored both the asymmetric static and time-varying volatility shock transmission mechanism across different volatility regimes. Therefore, prior studies have not taken into account whether the volatility shocks transmitted from EU-ETS towards the EU emerging economies equity markets are heterogeneous in the terms of persistence and magnitude across bullish quantiles (τ=0.95) as compared with the bearish (τ=0.05) and median (τ=0.50) quantiles.

In the existing literature, Luo et al. [61] investigated the predictability of equity market cycles in China by incorporating carbon transition risk as a key explanatory variable. The results indicated that carbon transition risk effectively accounts for fluctuations in equity market volatility, primarily through its influence on discount rates as a transmission mechanism. Suleman et al. [1] examined the symmetric transmission of shocks from the EU-ETS to the returns of European equity markets, utilizing the linear connectedness model developed by Diebold and Yilmaz [15]. The study identified France, Germany, and European sustainability indices as the primary sources of return spillovers, whereas carbon prices were found to have the minimal contribution. Wen et al. [2] investigated the non-linear linkages between Chinese ETS and equity returns and these asymmetrical linkages existed because of the more intensified effects of positive changes in the carbon trading market towards the equity returns. However, findings suggested insignificant shocks transmitted from stock returns towards the Chinese carbon trading market. To examine the asymmetric volatility spillovers between Chinese carbon trading and stock returns, Wu and Jiang [8] separated the aggregated equity and carbon volatilities into positive and negative volatility components using the GJR-GARCH method. Moreover, findings suggest that bad volatility spillovers transmit higher shocks as compared with good volatility thresholds and this adverse volatility shock transmission is enhanced in the wake of financial distress regime. Wu et al. [7] studies the symmetrical volatility spillovers between Chinese firms relying upon the higher carbon energy sources and omitting higher carbons and firms with low carbon intensity. Overall findings suggested that volatility spillovers from higher carbon firms are transmitted with greater intensity towards the lower carbon firms, moreover, the volatility spillovers between only higher carbon firms are also greater as compared with lower carbon firms.

Furthermore, environmentally responsible firms demonstrate superior performance compared to their less sustainable counterparts, yielding an annual excess return ranging from 6% to 7% despite a marginally elevated risk. These findings suggest that regulatory mechanisms, such as carbon allowance programs, significantly influence the risk-return dynamics of stocks, thereby encouraging investment in emission-conscious enterprises [62]. Xu et al. [6] investigated the transmission effects between the stock returns of carbon-intensified sectors and China’s carbon trading market using multifractal analysis, detrending methods, and time-lagged detrended cross-correlation approaches. The findings revealed positive cross-correlations in the Shenzhen and Shanghai pilots, while negative correlations were observed in the Beijing, Guangdong, and Hubei pilots. Additionally, the carbon trading market exhibited higher volatility and inefficiency compared to the stock market. Niu and Cao [63] explored the unequal interconnection between China’s carbon trading market and new energy stocks using the cross-quantilogram technique. The analysis identified modest yet notable bidirectional spillovers, characterized by varying correlation patterns among carbon markets. Furthermore, during periods of market decline, the new energy stock market emerged as a source of short-term extreme risks for Guangdong’s carbon market, while exerting a more pronounced influence on Hubei’s carbon market over extended lag periods. Zhao et al. [43] employed the frequency and time domain connectedness method proposed by Baruník and Křehlík [64] and Diebold and Yilmaz [15] to assess both the long- and short-term frequency domain as well as overall interconnections between energy-related commodities, GCC equity markets, and the carbon trading market. Overall findings suggested that both the energy related commodity, i.e., natural gas and emission trading system contributed the least error variances in the overall shock spillover mechanism.

Therefore, building upon the above existing studies, no effort is made to explore the quantile domain volatility shock transmission from EU-ETS towards the European emerging stock markets. Therefore, prior studies have not highlighted the heterogeneous and varied impact of the EU-ETS volatility shocks towards the EU emerging member economies stock volatility during bearish, bullish and moderate quantiles. For instance, Luo et al. [61] only emphasized on the effect of carbon related transition risk towards the Chinese stock markets, whereas, Wen et al. [3] analyzed the response of average value of the Chinese stocks to the negative and positive shocks in the Chinese carbon trading system. In a similar manner, Wu et al. [7] also utilized the mean centric granger causality VAR based approach for exploring the shock spillovers between Chinese carbon trading system and stock returns. Therefore, prior studies mostly take into account the Chinese equity markets in order to higher the shock spillovers from the carbon trading system in a linear and symmetrical fashion and ignored the heterogeneous dynamics of the bearish, bullish and moderate volatility regime specific shock spillovers. For instance, Xu et al. [6] also analyzed the correlational characteristics between Chinese carbon trading and stock returns and Niu and Cao [63] analyzed the dependence structures between sustainable stocks and carbon trading market within the Chinese context. whereas, [43] explored the shock spillovers between GCC financial asset classes and carbon trading system by using the mean centric GVAR basic connectedness approach by Diebold and Yilmaz [15]. However, the possibility of non-homogenous volatility shocks spillovers between EU-ETS and EU emerging stocks across bearish, bullish and moderate volatility regimes have not been explored. This poses the motivation for the observation of the quantile specific volatility interaction between EU-ETS and EU emerging economies stock markets.

Furthermore, Ahmed et al. [65] utilized the symmetrical VAR based connectivity approach in order to explore the higher order statistical moments of carbon trading market and energy related commodity markets. Overall findings suggested the increases in higher order statistical moments between all variables during the event of uncertainty. Furthermore, findings also suggested that the analysis of higher order statistical moments like excess kurtosis is relevant because of the presence of non-normalcy in financial time series observations. also utilized the symmetrical VAR domain connectivity approach in order to observe the spillover of shocks between sustainable bond indices of developed economies. Overall findings suggested that there exit time-varying opportunities for hedging the green bond indices because of the possibly lower spillover connectedness between them during few points of time in between 2008–2021. Chun et al. [66] observed the response of sustainable stock prices to the fluctuations in the coal and carbon prices by using the symmetrical GVAR based connectivity approach by Diebold and Yilmaz [15]. Overall findings suggested the adverse effect of coal prices on the renewable energy stocks, but opposite positive association existed between carbon intensive fuel and sustainable stock returns. Whereas, Bouri [67] provided new inferences for policy makers by exploring the mean domain symmetrical connectedness between conventional (brown) and sustainable (green) energy indices and technological stocks. The overall TVP-VAR based connectivity approach suggested that sustainable energy indices played a contributory factor in transmitting the volatility and excess kurtosis shock spillovers towards all others. Dutta et al. (2018) also employed the linear VAR based shock spillover mechanism in order to understand the volatility linkages between carbon trading system and sustainable stocks. Overall findings indicated the insignificant return-based shock spillovers but volatility shock spillovers between carbon trading and renewable stocks are higher in magnitude and persistent. Furthermore, the findings indicated that the overall volatility shock spillover are country specific because of the insignificant connectivity between carbon and stock markets of U.S. However, prior studies mostly take into account the effect of carbon emission trading system on the stock returns, but Marín-Rodríguez et al. [68] explored the impact of green bond indices on the carbon trading market and energy related commodities in a symmetrical manner. Overall findings suggested the adverse impact of carbon market on the sustainable bond index. Furthermore, also highlighted that quantile domain econometric estimation techniques are more efficient in the presence of non-linearity and structural breaks in data. Whereas, Dutta et al. [69] analyzed the fact that the volatility persistence of carbon trading cost has significantly decreased in the presence of structural breaks. Furthermore, when these structural breaks are incorporated into the forecasting model, the impact of both positive and negative shocks on the volatility of the carbon market increases. Overall, the results show that volatility persistence is overstated when structural breakdowns in the emission price risk are disregarded. This furthermore motivates us to explore the quantile domain shock transmission mechanism between EU-ETS and EU member economies’ stock market volatility.

This study significantly advances the existing literature on carbon stock nexus through taking a transition from mean centric GVAR based symmetric connectivity approach to asymmetric quantile-dependent volatility dynamics, which takes into account whether the EU-ETS affects developing European equity markets in a heterogeneous manner under bearish, bullish and moderate volatility regimes. Therefore, fund managers may effectively devise quantile domain hedging strategies for financial asset classes and diversify during bearish vs bullish phases of EU-ETS through a proper analysis of the non-homogenous nature of the EU-ETS and stock market volatility. The higher spillovers in bullish volatility regime suggest that carbon and stock market portfolios require higher hedging efficacy when both carbon and stocks are under stress (bullish volatility regime). This is classified as a situation that is predominantly ignored in existing literature. Therefore, the mean centric econometric approaches like generalized vector auto regression take into account the implicit assumption that volatility shock propagation is symmetric, homogenous, and stable across the return distribution [34,35,42,54,55]. This research article contributes to the existing literature by demonstrating that that shock propagation is quantile dependent and heterogeneous across quantiles than homogenous by using quantile-domain modeling. This demonstrates how shocks’ transmission mechanism from EU-ETS to the equity markets proliferate in a heterogeneous manner during bearish and bullish volatility regimes. This aligns with the finding of the existing studies relying upon the financial contagion theory by finding the quantile-specific risk behavior between financial asset classes [5]. The mean centered Generalized VAR based connection strategy just records the average reaction, ignoring tail occurrences when the fat-tailed, nonlinear, or asymmetrical behavior of EU-ETS and stock returns is dominating. As a result, portfolio managers may create carbon-equity portfolios that adjust exposure according to market conditions. When carbon-price volatility disproportionately affects emerging EU equity markets at specific quantiles, policymakers should employ stabilizing mechanisms or focused communication during risk periods (bullish volatility regime) to prevent unanticipated financial spillovers. Mean-based connectedness approaches are unable to identify quantile-dependent spillover intensification, which traders can exploit through spread trading, volatility arbitrage, and volatility regime-switching strategies. Thus, this research article conceptually advances the field by showing that relying just on mean-effects obfuscates the “true” nature of volatility spillovers, especially under stress or exuberance.

The term utilized in this research article as “Asymmetry” in the quantile domain between the EU-ETS and emerging European stock-market volatility explains whether volatility shock transmission differs greatly across higher, lower, and median quantiles instead of diffusing in a uniform manner across the entire return distribution. This is generally due to the fact that carbon and stock markets are already under stress in different volatility regimes like bullish (higher quantiles) and bearish (lower quantiles), thereby the connectedness indices between the both may be muted or absorbed differently [5]. In contrast, the carbon and stock market volatility shocks are transmitted with higher magnitude and persistence in upper quantiles (bullish regimes) because of decreased investment initiatives by the traders and portfolio optimizers. Huber et al. [70] also suggested that investors are less willing to invest in the riskier stocks. This state-dependent behavior is indicative of “regime-shift asymmetry”, which classifies that when the carbon market moves from the lower volatility state towards the higher volatility regime, the structural mechanism by which EU-ETS volatility influence the equity market is fundamentally transited. However, interconnection is dynamic and constantly changing in reaction to changes in energy costs, financial cycles, changes in carbon policy, and geopolitical upheavals and this is recognized as the “quantile-based time-varying dynamics of shock spillovers”. This furthermore provides the ample justification regarding the utilization of quantile-based spillover of shocks as compared with the mean-based connectivity approach and these are necessary to reveal the actual shape of EU-ETS–equity volatility transmission.

## 3. Data with descriptive statistics

### 3.1. Data

The research employs daily time series data covering the period from January 1, 2014, to December 1, 2024. It includes stock market indices from Central European emerging economies (Hungary, Greece, the Czech Republic, Slovakia, and Poland) as well as price data from the European Union Emissions Trading System (EU-ETS). The data sets are obtained from the London Stock Exchange Group’s data analytics platform (https://www.lseg.com/en/data-analytics) and also accessed via the Bloomberg terminal. This study draws upon the MSCI classification to identify the Czech Republic, Hungary, Greece, Slovakia, and Poland as key European central emerging stock markets. Additionally, the Visegrad Group (V4) represents an informal regional alliance comprising four Central European nations—Poland, the Czech Republic, Slovakia, and Hungary—linked by their geographical proximity, shared historical experiences, and common cultural heritage and values [71]. The Czech Republic, Hungary, and Poland have also been selected as representatives of Central European emerging equity markets to explore their long-term co-integration with developed financial markets. Azimli [72] investigated how firm-level capital investment in these Central European emerging markets (Czech Republic, Hungary, and Poland) responds to fluctuations in economic policy uncertainty. The findings indicate that these economies demonstrate resilience to disruptions in economic policy. Similarly, Czapkiewicz et al. [73] analyzed the relationship between European emerging equity markets (Czech Republic, Greece, Hungary, and Poland) and idiosyncratic volatility, revealing that cross-sectional returns are adversely influenced by idiosyncratic volatility. Syriopoulos [36] have explored the influence of monetary policy on the long-term integration between developed equity markets of U.S. with the selected European emerging equity markets, i.e., Poland, Czech Republic, Hungary, Slovakia.

Moreover, in order to estimate the conditional volatility of European carbon emission trading system (EU-ETS) and European central emerging economies’ stock returns of Czech Republic, Greece, Hungary, Poland and Slovakia, we take into account the generalized conditional heteroscedasticity modelling framework (GARCH) by [74]. The EU-ETS and European central emerging economies stock returns are estimated as $r_{t}=Ln\;\left(\frac{P_{t}}{P_{t-\text{1}}}\right)$. The mean equation for the GARCH (1,1) approach can be estimated as: $r_{t}=\mu +\varepsilon_{t\;,}$ whereas, the $\varepsilon_{t\;}$ can be estimated as $z_{t}\sigma_{t}$ and $z_{t}$ is classified as the standardized error term ($z_{t}\sim N(\text{0},\text{1}))$. The variance equation for the GARCH (1,1) approach can be estimated as: $\overset{\text{2}}{\underset{t}{\sigma }}=\omega +\alpha \overset{\text{2}}{\underset{t-\text{1}}{\varepsilon }}+\beta \overset{\text{2}}{\underset{t-\text{1}.}{\sigma }}$ Therefore, $\overset{\text{2}}{\underset{t}{\sigma }}$ is classified as the variance (risk) of the EU-ETS and European central emerging economies’ stock returns. In recent scholarly work, Shahbaz et al. [75] utilized the univariate GARCH methodology to derive the conditional risk series associated with industrial metals. This approach was utilized to investigate the interconnectedness between these metals and factors such as financial stress, commodity market uncertainty, and climate-related policies.

The time span from January 2014 to December 2024 is selected due to several key factors. First, Phase III of the Emissions Trading System (ETS) reform, initiated in 2013, introduced transformative changes, including a single EU-wide emissions cap replacing national caps and a shift from free allocation to auction-based distribution allowance. Second, the European debt crisis (2009–2014) caused significant financial turmoil in the Eurozone due to high government debt, fiscal imbalances, and economic vulnerabilities. Third, the quantile-domain shock transmission mechanism between the stock markets of European central emerging economies and EU-ETS volatility captures pivotal events such as Brexit (2016), the COVID-19 pandemic (2019–2022), the Russia-Ukraine war (2022), and recent geopolitical tensions in the Israel-Gulf economies. Additionally, this period for the analysis of this study spanning over decade includes developments like the Market Stability Reserve (MSR) introduction in 2015 and implementation in 2019, the resulting surge in EU-ETS prices, the Paris Agreement in 2015, and the Carbon Border Adjustment Mechanism rollout in 2023. Analyzing shock transmission during these years provides crucial insights for stakeholders across economic and business cycles.

### 3.2. Descriptive statistics

**Fig 1:**
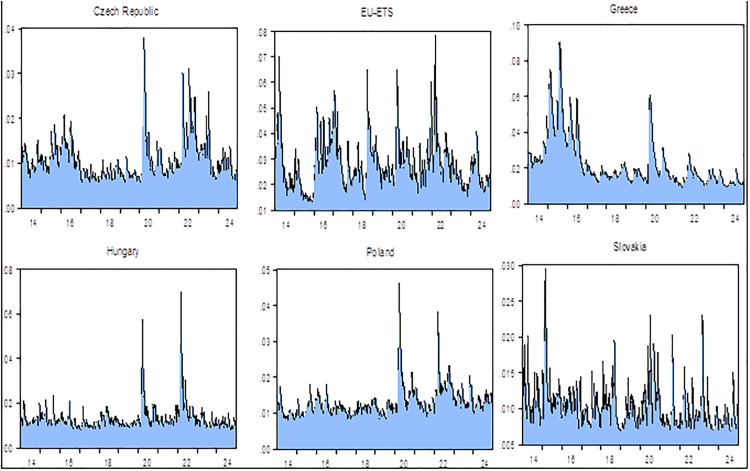
The graphical representation of the conditional volatility series of European emerging economies’ stock markets and EU-ETS.

Table 1 indicates that among European emerging economies, Greece, Poland, and Hungary exhibited the highest average conditional volatilities, with values of 0.0217, 0.0126, and 0.0123, respectively, surpassing those of Slovakia and the Czech Republic. Likewise, the EU-ETS reported a significantly higher average conditional volatility of 0.0267 compared to the equity returns of other European emerging countries. Fig 1 illustrates that the conditional volatility series for equity markets across all European emerging economies showed significant upward fluctuations between 2014 and 2016, during the COVID-19 period, from 2022 to 2023, and throughout 2024. Similarly, Fig 1 also illustrates that conditional volatility in the EU-ETS increased significantly during 2014, between 2016 and 2017, and notably surged during 2018 and the COVID-19 period. Additionally, Fig 1 also highlights that heightened volatility was also evident in the conditional volatility of the EU-ETS during 2022 and 2024. The European Parliament report highlights that the volatility in both the stock and EU-ETS markets escalated between 2014 and 2016, driven by the prolonged impact of the European debt crisis and uncertainties surrounding Greece’s economic stability [76]. The 2014 annexation of Crimea by Russia and the subsequent conflict in Ukraine significantly increased market volatility in Eastern Europe [77], with the migrant crisis exacerbating instability in Hungary and Poland. The heath crisis regime led to significant disruptions in 2020 [78], resulting in increased volatility within the European emerging stock markets and the EU-ETS (Suleman, Rehman, et al., 2023). The 2022 Ukraine war escalated geopolitical risks, energy price fluctuations, and inflation, worsening instability and thereby causing an upsurge in the conditional volatility series of European emerging economies’ stock markets and EU-ETS. By 2024, volatility remained elevated due to ongoing inflation, increasing interest rates, and uncertain economic growth, further aggravated by the Ukraine-Russia conflict and rising geopolitical tensions involving Israel, the U.S., and the Middle East. Thus, investigating the extreme shock transmission mechanism within the quantile framework between stock volatility in European emerging economies and the EU-ETS during these time periods offers valuable insights for enhancing stock and EU-ETS volatility hedging strategies and optimizing portfolio returns.

**Table 1:** Descriptive statistics of the conditional volatility series of MSCI European emerging economies’ stock markets and European Union Carbon Emission Trading System (EU-ETS).

Descriptive Statistics	Czech Republic	EU-ETS	Greece	Hungary	Poland	Slovakia
Mean	0.010254	0.026706	0.021707	0.012383	0.012633	0.009731
Median	0.009052	0.02497	0.01681	0.011147	0.011697	0.009113
Maximum	0.03806	0.078235	0.0906	0.069603	0.046466	0.029478
Minimum	0.005459	0.012682	0.00886	0.00665	0.007801	0.00677
Std. Dev.	0.004329	0.00917	0.013373	0.005161	0.004128	0.002565
Skewness	2.248342	1.53412	2.097382	5.229636	3.370963	2.108757
Kurtosis	10.05113	6.443776	7.736748	40.84215	20.57666	10.43459
Jarque-Bera	8255.677	2511.182	4725.54	181952.4	41833.07	8624.185
Probability	0.0000	0.0000	0.0000	0.0000	0.0000	0.0000
Sum	29.04954	75.65904	61.49721	35.07999	35.78888	27.56819
Sum Sq. Dev.	0.05308	0.238129	0.506461	0.075446	0.048263	0.018633
Observations	2833	2833	2833	2833	2833	2833
Unit root test at level						
ADF	−6.14***	−6.19***	−3.82***	−8.102***	−7.77***	−10.41***
PP	−6.182***	−6.745***	−3.65***	−7.68***	−7.726***	−10.36***

Note: This table presents the descriptive statistics for the conditional volatility of the European Union Carbon Emission Trading System (EU-ETS) and the stock returns of emerging economies in the European Union, including the Czech Republic, Slovakia, Greece, Hungary, and Poland. The final section of the table provides the results of the Augmented Dickey-Fuller (Dickey & Fuller, 1981) and Phillips-Perron (Phillips & Perron, 1988) unit root tests for the conditional volatility series of the EU-ETS and the stock markets of these emerging economies.

Furthermore, the conditional volatility series for the EU-ETS and European developing economies are leptokurtic, indicating the existence of severe outliers in the dataset, according to the descriptive statistics as shown in Table 1. This significant variability highlights the suitability of using a quantile-based Vector Auto-regression (QVAR) framework over conventional mean-based VAR connectedness technique. It is typified by abrupt upward and downward movements in the conditional volatility series and the non-normality of the data. When examining datasets with leptokurtic distributions and departures from normality, the QVAR technique is especially well-suited for capturing asymmetries and tail dependencies [17]. Fig 1 also show that there are frequent upside and downside fluctuations in both the EU-ETS and EU emerging economies stock market volatility during the notable events like COVID-19, geopolitical uncertainty amid Russian-Ukraine war and during the trade policy uncertainty as U.S. imposes trade restrictions on China in 2018. These frequent fluctuations from the mean values may be indication of leptokurtic data distribution and presence of extreme outliers [16,19]. Therefore, [5] also classified that in the presence of uncertainty events, the utilization quantile domain shock spillover mechanism is warranted as there may be the higher existence of structural breaks, excess kurtosis and non-normalcy of data. Furthermore, the results showthat the null hypothesis of data independence and identical distribution (*i.i.d.)* is rejected for the conditional volatility series of EU-ETS and EU emerging economies stock markets (see Appendix Table A1 in S1 Appendix). The rejection of null hypothesis of independence and identical distribution implied the presence of asymmetry in the time-series data, making the estimates from the traditional mean-based VAR models biased (see Appendix Table A1 in S1 Appendix). Therefore, the estimation of the QVAR is technically appropriate as quantile specific VAR approach imposes no assumption of linearity or *i.i.d.* errors. The QVAR model is dependent across conditional quantiles, thereby causing the autoregressive structure to differ at varied distributional points. This feature enables QVAR to capture quantile dependent nonlinear connectedness and tail-specific volatility shock spillovers between EU-ETS and EU emerging stocks.

Table 1 further illustrates that the conditional volatility series of stock markets in European emerging economies and the EU-ETS are integrated at the same order, specifically *I* (0). The findings indicate that all variables are stationary, as validated by the Phillips-Perron [79] and Augmented Dickey-Fuller [80] tests for unit roots. Therefore, the use of the quantile VAR method is justified for investigating the risk transmission dynamics between the stock market volatility and EU-ETS at various quantile levels.

## 4. Methodology

For the purpose of exploring the extreme shock spillovers between EU-ETS and emerging economies’ stock volatility, a quantile-based vector auto-regression (QVAR) model incorporating the “Extended Joint” volatility spillover mechanism proposed by Cunado et al. [38] is employed. As highlighted by Sheikh et al. [17], lower quantile, such as τ = 0.05, signify periods of bearish stock and EU-ETS market volatility, whereas higher quantile, such as τ = 0.95, correspond to bullish stock market and EU-ETS volatility conditions The selection of quantiles (τ = 0.05, 0.50, and 0.95) aligns with the approach adopted by Iacopini et al. [61] and Long and Li [81].

One of the major justification that Quantile domain VAR (QVAR) based connectedness approach by Ando et al. [40] is more suitable than mean based connectivity approach is that the volatility shock transmission between EU-ETS and stock markets is mainly concerted in the tails of the conditional distribution due to the presence of leptokurtic distribution, presence of non-linearity and non-normalcy of the data. The volatility shock spillovers in the presence of leptokurtic data distribution, non-normalcy and non-linearity cannot be captured by mean-driven connectivity approaches based upon the generalized VAR approach (see Diebold and Yilmaz [15]. As contrast to QVAR approach, the standard VAR models only take into account the shock transmission process during the average market conditions due to its reliance on the mean-based process. Therefore, mean based connectivity approaches imposes symmetry, linearity, and distributional homogeneity across all market states [36,63]. Subsequently, the QVAR approach can incorporate the heterogeneous shock spillovers between EU-ETS and EU emerging economies’ stock volatility that usually take place during extreme carbon-price shocks, financial stress episodes, or abrupt volatility clustering. These features are extremely dominant in carbon and financial asset classes [16,19,24]. However, the QVAR driven connectivity approach relied upon the conditional quantile function, $Q_{\tau }(y_{t}|y_{t-\text{1}})$, allow the slope coefficients to vary across bearish, bullish and moderate volatility regimes. This permits the identification of state-dependent spillovers [49]. This capability to explore shock spillovers across different volatility regimes enables the QVAR to distinguish stronger carbon-to-equity volatility transmission in lower or upper tails, where risk sensitivity is highest, while maintaining robustness to non-Gaussianity, heteroscedasticity, and heavy-tailed innovation. These are the possessions that traditional VAR cannot accommodate.

### 4.1. The Quantile based Vector Auto-regression (QVAR) approach with “Extended Joint” connectedness framework

To quantify the interconnectedness measures between conditional volatility in European emerging stock markets and the EU-ETS, we begin by estimating a quantile vector auto-regression model, referred to as QVAR(p). This methodology is outlined in detail by White et al. [82] and can be succinctly described as follows:


$$\begin{array}{c} y_{t}=\;\mu (\tau )+\;\sum_{j=\text{1}}^{p}\Phi_{j}(\tau )y_{t-j}+\mu_{t}(\tau ) \end{array}$$ (1)

Equation (1) uses K × 1 vectors for endogenous variables, denoted by $y_{t}$ and $y_{t-j}$. The symbol τ indicates a specific percentile (from 0 to 100) within a statistical distribution, representing the data’s percentile position. The lag length in the QVAR model is given by p. The K × 1 conditional mean vector is represented by **μ**(τ) and is the K × K QVAR coefficient matrix. The K × 1 error vector is $\mu_{t}(\tau )$, with a K × K variance-covariance matrix ***Σ***(τ). T represents the time dimension. The World theorem can be applied to extend the QVAR(p) model to a QVAR (∞) framework.


$$\begin{array}{c} y_{t}=\;\mu (\tau )+\;\sum_{j=\text{1}}^{p}\Phi_{j}(\tau )y_{t-j}+\mu_{t}(\tau )=\mu (\tau )+\;\sum_{i=\text{0}}^{\infty }A_{i}(\tau )\mu_{t-i}(\tau ) \end{array}$$ (2)

Subsequently, we calculate the “generalized forecast error variance decomposition” (GFEVD) over a time horizon extending *H* steps into the future. This approach, introduced by Koop et al. [83] and further refined by Pesaran and Shin [84], facilitates the analysis of the influence of a disturbance originating in series *j* on series *i*. In this framework, the vector $e_{i}$ is defined as a unit vector with all elements equal to zero except for the value of one at the $i^{th}$ position.


$$\begin{array}{c} \overset{gen}{\underset{ij}{\psi }}(H)=\;\frac{{\sum \overset{-\text{1}}{\underset{ii}{\left(\tau \right)}}\sum_{h=\text{0}}^{H-\text{1}}\left(e^{\prime }A_{h}(\tau )\sum (\tau )e_{j}\right)}^{\text{2}}}{\sum_{h=\text{0}}^{H-\text{1}}\left({e_{i}}^{\prime }A_{h}(\tau )\sum (\tau )A_{h}(\tau {)}^{\prime }e_{i}\right)} \end{array}$$ (3)

$$\begin{array}{c} {gSOT}_{ij}(H)=\frac{\overset{gen}{\underset{ij}{\psi }}(H)}{\sum_{j=\text{1}}^{K}\overset{gen}{\underset{ij}{\phi }}(H)} \end{array}$$ (4)

Moreover, the overall influence of all other markets on a specific market i (referred to as “FROM” connectedness), and the influence of market i on the overall network (“TO” connectedness), can be defined as follows:


$$\begin{array}{c} \overset{gen,FROM}{\underset{all\to i}{S}}(H)=\;\sum_{j=\text{1},i=\;\;/\;j}^{K}{gSOT}_{ij}(H) \end{array}$$ (5)

$$\begin{array}{c} \overset{gen,TO}{\underset{i\to all}{S}}(H)=\;\sum_{j=\text{1},i=\;\;/\;j}^{K}{gSOT}_{ji}(H) \end{array}$$ (6)

The net shock transfer is determined by the difference between the sum of shocks transmitted from a variable (“i”) to all other variables (“j”) and the total shocks “i” receives from those same variables “j”. This can be represented mathematically as: $\overset{gen,NET}{\underset{i}{S}}(H)=\overset{gen,TO}{\underset{i\to all}{S}}(H)-\overset{gen,FROM}{\underset{all\to i}{S}}(H)$. A positive result signifies that “i” has a greater influence on the other variables than they have on “i”. A negative result indicates the opposite – “i” is more affected by the others. Therefore, “i” is considered a net shock transmitter if the result is positive, i.e., $\overset{gen,NET}{\underset{i}{S}}(H)$ > 0 and a net shock receiver if negative, i.e., $\overset{gen,NET}{\underset{i}{S}}(H)$ < 0 (referencing [1,85]. A key metric within this connectedness analysis is the Total Connectedness Index (TCI). This index quantifies the level of interconnectedness within the system and its importance for understanding market risk. The TCI reflects the average overall directional influence both sent to and received from other parts of the system and has the following mathematical definition: $gSOI(H)\;=\;\frac{\text{1}}{k}\sum_{i\;=\;\text{1}}^{k}\overset{gen,from}{\underset{all\to i}{S}}(H)\;=\;\frac{\text{1}}{k}\sum_{i\;=\;\text{1}}^{k}\overset{gen,TO}{\underset{i\to all}{S}}(H)$. Additionally, the net pairwise dynamic connectedness (NPDC) can be calculated as: $\overset{gen,net}{\underset{i,j}{S}}(H)\;=\;\overset{gen,TO}{\underset{ji}{gSOT}}(H)-\overset{gen,From}{\underset{ij}{gSOT}}(H)$.

#### 4.1.1. QVAR with extended joint connectedness measure.

A critique by Caloia et al. [86] highlighted potential inaccuracies in the standard normalization procedure used in typical generalized vector autoregressive (GVAR) interconnectedness analyses, such as those developed by Diebold and Yilmaz [15]. The authors suggested this method could lead to flawed conclusions about how disturbances spread through the system (Sheikh et al., 2024). Lastrapes and Wiesen [87] offered an alternative, the “Joint” spillover index, which emphasizes model fit. Further development by Balcilar et al. [41] involved merging the techniques of Lastrapes and Wiesen [69] with those of Antonakakis et al. [88] within a Time-Varying Parameter Vector Auto-regression (TVP-VAR) context, creating the “extended joint” TVP-VAR interconnectedness model. Later, Cunado et al. [38] integrated the established quantile-based vector auto-regression (QVAR) approach of Ando et al. [40] with the “extended joint” methodology from Balcilar et al. [41], producing the “extended joint” QVAR framework, which provided a more sophisticated version of the standard QVAR-based interconnectedness analysis.

Balcilar et al. [37] presented an improved interconnectedness technique, termed the “extended joint” method, which effectively addresses issues stemming from normalizing by row sums. Implementing this technique within a quantile vector autoregressive (QVAR) structure produces results consistent with the “Joint Spillover” approach proposed by Lastrapes and Wiesen [69], facilitating the computation of Net Pairwise Directional Connectedness (NPDC) measures [37]. By employing an enhanced normalization technique, the extended joint QVAR method improves the accuracy of connectedness estimates [20]. In contrast to conventional, symmetric vector autoregressive (VAR) methods for analyzing shock transmission, this enhanced QVAR methodology decomposes forecast error variance by examining the combined effects of shocks from other variables across different points of the distribution (τ=0.05, 0.50 and 0.95). As a result, the spillover metrics generated by the “extended joint” QVAR framework provide a more nuanced and thorough understanding of spillover patterns than standard QVAR techniques [38].

The mathematical notation $\overset{Joint,From}{\underset{all\to i}{S}}(H)$ characterizes the impact that all variables within the network exert on series i. The precise expression for this in mathematical way is as follows:


$$\begin{array}{c} \xi_{\text{1}}(H)=y_{t+H}-E\left(y_{t+H}|y_{t,}y_{t-\text{1},\ldots \ldots .}\right)=\sum_{h=\text{0}}^{H-\text{1}}A_{h}\epsilon_{t+H-h} \end{array}$$ (6)

$$\begin{array}{c} {E(\xi }_{\text{1}}(H){(\xi^{\prime }}_{\text{1}}(H))=A_{h}\sum {A^{\prime }}_{h} \end{array}$$ (7)

$$\begin{array}{c} \overset{Joint,From}{\underset{all\to i}{S}}(H)=\frac{{E(\xi^{\text{2}}}_{i,t}(H))-E[\xi_{i,t}(H)-E(\xi_{i,t}(H))|\epsilon_{\forall =\;\;/\;i,t+\text{1},\ldots \ldots }{\epsilon_{\forall =\;\;/\;i,t+H}]}^{\text{2}}}{{E(\xi^{\text{2}}}_{i,t}(H)} \end{array}$$ (8)

$$\begin{array}{c} =\frac{\sum_{h=\text{0}}^{H-\text{1}}\overset{\prime }{\underset{i}{e}}A_{h}\sum M_{i}({\overset{\prime }{\underset{i}{M}}\sum \overset{\prime }{\underset{i}{M}})}^{-\text{1}}\overset{\prime }{\underset{i}{M}}\sum \overset{\prime }{\underset{h}{A}}e_{i}}{\sum_{h=\text{0}}^{H-\text{1}}\overset{\prime }{\underset{i}{e}}A_{h}\sum \overset{\prime }{\underset{h}{A}}e_{i}} \end{array}$$ (9)

Here, $M_{i}\;$ represents a matrix with dimensions of K × (K − 1), essentially derived from an identity matrix by removing the $\;i^{th}$ column. The vector ϵ∀ =/ i, t + 1 signifies the set of unexpected changes at a given time t + 1  for all series excluding series i , and its size is K − 1. We then proceed to compute the “Joint” TCI using the following formula.


$$\begin{array}{c} jSOI(H)=\;\frac{\text{1}}{K}\sum_{i=\text{1}}^{k}\overset{Joint,From}{\underset{all\to i}{S}}(H) \end{array}$$ (10)

This falls within a scale from 0 to 1, differing from the original Total Connectedness Index (TCI) approach, as highlighted by Chatziantoniou et al. [89] and Gabauer [90]. Building on their previous work, Balcilar et al. [41] incorporated several scaling factors to establish a link between *gSOT* and *jSOT* [21].


$$\begin{array}{c} \lambda_{i}(H)=\;\frac{\overset{Joint,From}{\underset{all\to i}{S}}(H)}{\overset{gen,From}{\underset{all\to i}{S}}(H)} \end{array}$$ (11)

$$\begin{array}{c} {jSOT}_{ij}(H)=\lambda_{i}(H)gS{OT}_{ij}(H) \end{array}$$ (12)

Equations (13), (14), and (15) serve to quantify the total directional influence of a specific variable i on all other variables (“TO” connectedness), along with the “NET” total directional impact (the difference between “TO” and “FROM” connectedness) and the net pairwise directional connectedness (NPDC). These calculations are then utilized to create network visualizations within the quantile domain.


$$\begin{array}{c} \overset{Joint,TO}{\underset{i\to all}{S}}(H)=\;\sum_{j=\text{1},i=\;\;/\;j}^{K}{jSOT}_{ji}(H) \end{array}$$ (13)

$$\begin{array}{c} \overset{Joint,NET}{\underset{j}{S}}(H)=\;\overset{Joint,TO}{\underset{i\to all}{S}}(H)-\overset{Joint,FROM}{\underset{all\to i}{S}}(H) \end{array}$$ (14)

$$\begin{array}{c} \overset{Joint,NET}{\underset{ij}{S}}(H)=\overset{Joint,TO}{\underset{ji}{jSOT}}(H)-\overset{Joint,FROM}{\underset{ij}{jSOT}}(H) \end{array}$$ (15)

### 4.2. The hedge ratio strategy and optimal portfolio weight selection approach

Adopting the analytical framework of Sheikh and Suleman [91], this research contrasts a hedging strategy based on hedge ratios with a method for selecting optimal portfolio weights to ascertain which risk mitigation approach is superior in reducing risks stemming from long-term volatility in the EU-ETS or instability in European emerging stock markets. Portfolio analysis is conducted using a Dynamic Conditional Correlation Generalized Autoregressive Conditional Heteroskedasticity (DCC-GARCH) model incorporating a student’s t copula. Dynamic hedge ratios are computed following the approach of Kroner and Sultan [92], while optimal portfolio weights are established using the technique devised by Kroner and Ng [42]. The hedge ratio strategy focuses on evaluating the effectiveness of managing long-term risks in the EU-ETS by utilizing short-term positions in European equity markets and vice versa. On the other hand, the optimal portfolio weight selection strategy aims to explore whether integrating European emerging equity markets into a portfolio can provide better protection against EU-ETS volatility. Furthermore, as per the methodology proposed by Shahbaz et al. [93], the econometric framework introduced by Ederington (1979) is applied to measure hedging effectiveness (HE). The estimation of hedge ratios and optimal portfolio weights is conducted using the DCC-GARCH-t copula approach, consistent with the work of Shahbaz et al. [75] and Tabash et al. [94]. The strategy for determining the hedge ratio, along with its associated hedging effectiveness (HE), can be evaluated using the following approach.


$$\begin{array}{c} \beta_{SI.EUETS,t}=\frac{h_{SI.EUETS,t}}{h_{SI,t}} \end{array}$$ (16)

$$\begin{array}{c} \text{HE }=\;\text{1}-\frac{VAR(r_{\beta })}{VAR(r_{unhedged})} \end{array}$$ (17)

In the given equation, ${(h}_{SI.EUETS,t})\;$ signifies the time-varying comovement between stock market values of developing European nations (SI) and the European carbon market (EU-ETS). Conversely, ${(h}_{SI,t})\;$ denotes the time-varying volatility of these developing European stock markets. The hedge ratio ($\beta_{SI.EUETS,t}\;in\;Eq.\text{1}\text{6})$ assesses whether a temporary investment strategy in European stock markets can optimally mitigate risk against sustained fluctuations in the European carbon market. However, to achieve optimal risk reduction against persistent changes in stock market values of developing European nations through a short-term trading strategy in the European carbon market, the hedge ratio calculation can also be reformulated as follows


$$\begin{array}{c} \beta_{EUETS.SI,t}=\frac{h_{EUETS.SI,t}}{h_{EUETS,t}} \end{array}$$ (18)

Equation 19 outlines the optimal portfolio weighting approach, which examines whether constructing a portfolio using returns from emerging European stock markets offers improved hedging performance and mitigates risk associated with EU-ETS volatility, and the reverse scenario. In the above equation, $h_{EUETS,t}$ and $h_{SI,t}$ are the conditional variances of EU-ETS and European emerging economies’ stock indices (SI). Whereas $h_{SI.EUETS,t}$ is the conditional co-variance between EU-ETS and European emerging economies’ stock indices (SI) at a time t.


$$\begin{array}{c} w_{SI.ETS,t\;}=\frac{h_{EUETS,t}-h_{SI.EUETS,t}}{h_{SI,t}-\;{\text{2}h}_{SI.EUETS,t}+h_{EUETS}},\text{ with }\overset{SI}{\underset{t}{w}}=\left\{ \begin{array}{cc} \text{0} & \overset{SI}{\underset{t}{w}}<\text{1}\\ \overset{SI}{\underset{t}{w}} & \text{0}\leq \overset{SI}{\underset{t}{w}}\leq \text{1}\\ \text{1} & \overset{SI}{\underset{t}{w}}>\text{1} \end{array}\right.\; \end{array}$$ (19)

## 5. Results with practical implications

**Fig 2:**
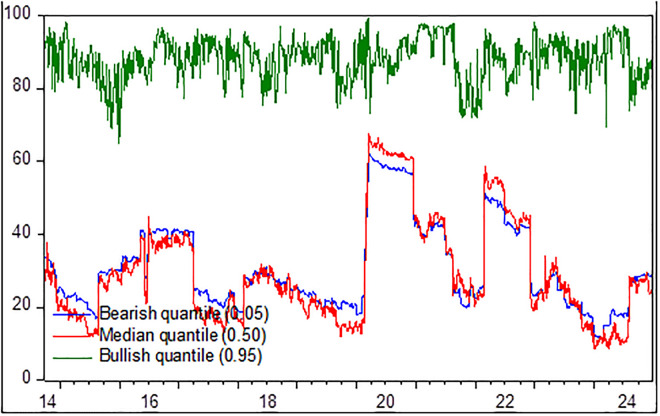
The “Extended Joint” QVAR based connectedness between EU-ETS and European emerging economies’ stock volatility.

**Fig 3:**
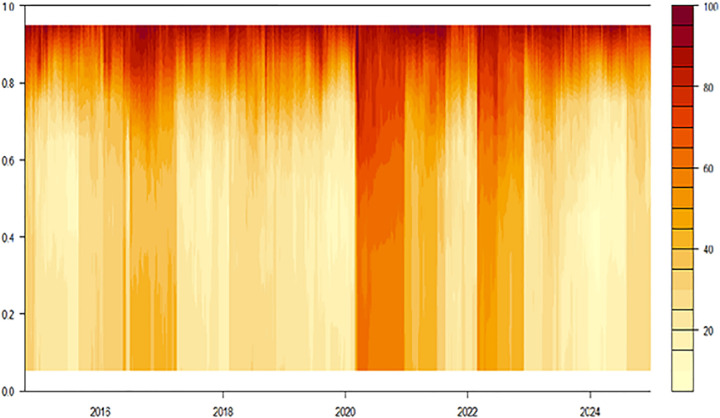
Graphical representation of the quantile domain volatility shock spillovers between EU-ETS and European emerging economies.

Fig 2 illustrates the overall volatility interconnectedness between the EU-ETS and MSCI European emerging stock markets at lower (τ = 0.05), median (τ = 0.50), and higher quantile (τ = 0.95). Fig 3 presents a heat map of this asymmetrical connectedness, showing that darker (lighter) shades indicate higher (lower) conditional volatility shock spillovers between the EU-ETS and equity markets at varied volatility conditions, i.e., bullish, bearish, and moderate conditions. Fig 2 reveals that overall volatility interconnectedness is more pronounced at higher quantile (τ = 0.95) compared to the median (τ = 0.50) and lower (τ = 0.05) quantiles. The pronounced EU-ETS and stock interconnectedness at higher quantiles underscores that, under extreme market conditions (characterized by a bullish volatility regime), fluctuations in EU-ETS volatility exert a considerable influence on European stock markets. The findings in Table 2 highlight critical insights for policymakers, risk managers, and market participants. The greater contribution of error variances in forecasting 20-day-ahead conditional risk in the EU-ETS at higher quantile (τ = 0.95) implies that volatility shocks in European emerging equity markets exert a disproportionately stronger influence under extreme bullish conditions. This suggests that during periods of heightened market volatile behavior, EU-ETS risk is more susceptible to external equity market volatility. Additionally, the significantly higher aggregated spillover measure (88.01%) during bullish volatility conditions compared to the median (29.43%) and bearish (30.63%) conditions underscores the asymmetric nature of interconnectedness.

**Table 2:** The quantile domain shock transmission mechanism between the conditional volatilities in the European emerging economies’ stock markets and carbon emission trading system (EU-ETS).

At lower quantiles (τ=0.05)	EU-ETS	Czech Republic	Greece	Hungary	Poland	Slovakia	FROM
EU-ETS	75.03	5.04	5.44	6.57	5.56	2.35	24.97
Czech Republic	4.68	65.75	7.88	9.51	10.59	1.6	34.25
Greece	5.27	8.02	68.66	8.08	8.71	1.27	31.34
Hungary	6.32	9.93	8.24	58.59	15.6	1.32	41.41
Poland	5.66	11.26	9.1	15.71	56.66	1.59	43.34
Slovakia	2.62	1.71	1.29	1.32	1.54	91.51	8.49
TO	24.55	35.96	31.95	41.19	42	8.13	183.79
Inc.Own	99.58	101.72	100.61	99.79	98.66	99.65	**TCI**
NET	−0.42	1.72	0.61	−0.21	−1.34	−0.35	**30.63**
**At median quantiles** (τ=0.50)	**EU-ETS**	**Czech Republic**	**Greece**	**Hungary**	**Poland**	**Slovakia**	**FROM**
EU-ETS	77.8	4.61	3.91	4.93	5.55	3.2	22.2
Czech Republic	2.79	67.69	8.14	9.62	10.33	1.44	32.31
Greece	5.02	7.63	66.29	9.17	10.63	1.27	33.71
Hungary	4.47	8.48	7.7	60.48	17.38	1.49	39.52
Poland	3.67	12.65	9	14.91	56.83	2.95	43.17
Slovakia	1.71	1.27	0.84	0.8	1.02	94.36	5.64
TO	17.65	34.65	29.6	39.42	44.9	10.34	176.55
Inc.Own	95.45	102.34	95.88	99.9	101.73	104.7	**TCI**
NET	−4.55	2.34	−4.12	−0.1	1.73	4.7	**29.43**
**At bullish quantiles** $\begin{array}{c} \;\left(\tau =0.95\right) \end{array}$	**EU-ETS**	**Czech Republic**	**Greece**	**Hungary**	**Poland**	**Slovakia**	**FROM**
EU-ETS	13.22	15.66	15.43	19.3	18.39	18	86.78
Czech Republic	19.37	10.55	15.37	18.98	18.5	17.23	89.45
Greece	18.9	15.6	9.96	19.48	18.3	17.76	90.04
Hungary	19.49	15.98	16.51	10.65	19.01	18.36	89.35
Poland	19.76	16.46	16.32	20.1	9.06	18.29	90.94
Slovakia	17.86	14.43	14.42	17.82	16.98	18.5	81.5
TO	95.37	78.13	78.05	95.68	91.18	89.65	528.07
Inc.Own	108.59	88.68	88.01	106.33	100.24	108.15	**TCI**
NET	8.59	−11.32	−11.99	6.33	0.24	8.15	**88.01**

Note: This Table explain the volatility shock spillovers between European Union Carbon Emission Trading System (EU-ETS) and MSCI EU emerging stock markets by using the “Extended Joint” Quantile Vector Auto regression (QVAR) approach by Cunado et al. (2023). The integration of the “Extended Joint” connectedness approach of [41] with the traditional QVAR approach by [40] offers advanced normalization techniques, robustness to outliers and efficient in addressing interpretability challenges. Furthermore, the selection of varied quantiles, i.e., bearish (τ=0.05), bullish (τ=0.95) and moderate (τ=0.50) is in line with the Iacopini et al. (2023), Sheikh et al. (2024) and Tabash, Sheikh, et al. (2024). Moreover, the selection of the lag length is according to the minimum Akaike Information Criterion (AIC) and consistent with the exiting studies (see Chatziantoniou et al., 2021). The Total Connectedness Indices (TCI) explains the aggregated value of the overall forecast error variances due to the total spillover of volatility shocks within the entire QVAR system across different quantiles. Furthermore, “TO” explains the overall shock transmission from a market “i” towards all other markets “j”, whereas “FROM” explains the overall shock reception by a specific market “i” from all other markets “j” across quantiles. Furthermore, the “NET” volatility shock spillovers explains the overall different between the shock transmitted towards all markets “TO” and the volatility shock received by a particular market “FROM” all others across quantiles. Moreover, the selection of the H-step ahead forecasting horizon is in line with the [38] and [89].

This asymmetrical volatility shock transmission between EU-ETS and EU emerging member economies’ stock market is also consistent with the findings of Liu et al. [56] as the overall findings highlighted the differential impact of carbon market shocks on the industrial stocks at the bullish quantiles. The higher volatility shock spillovers between EU-ETS and EU emerging member economies stock markets across bullish quantiles is consistent with the findings of Shang and Hamori [95] who have reported that overall shock spillovers between commodity and stock markets are higher during extreme volatility regimes. Whereas, Wang et al. [96] also observed that financial asset classes transmit higher shocks at the extreme quantiles as compared with the median quantile. Furthermore, the higher volatility shock spillovers at higher quantiles is also consistent with the findings of Sheikh et al. [17] as the study reported the fact that at extreme higher quantiles, financial asset classes receive higher uncertainty shocks as compared with the median quantiles. Furthermore, the higher volatility shock spillovers between EU-ETS and EU emerging member economies stock markets at the extreme higher quantile as compared with the median and bearish quantile resembles the presence of non-homogeneity in the volatility shock spillover. As, Wen et al. [3] also reported the asymmetrical shock transmission between Chinese stocks and carbon trading system as positive carbon trading market shocks lead towards a more stronger and persistent effect on the stock returns as compared with the negative shocks.

Fig 2 shows the time-varying volatility shock spillovers between EU-ETS and EU emerging stock markets across all the quantiles but these volatility shock spillovers increase during 2014–2016, 2019 and during the timeframe of 2022. Several factors have contributed to the heightened time varying shocks between the EU-ETS and European emerging stock markets. According to report by Asian Development Bank, the EU-ETS adopted a “back-loading” strategy, withholding 900 million carbon allowances to address oversupply and stabilize prices [97], which may lead towards the heightened EU-ETS and stock market volatility spillovers between 2014–2016. Whereas Fig 2 also shows that the overall volatility shocks spillovers between EU-ETS and EU emerging stock markets increases during 2014–2016 across all the quantiles. Following the 2016 Paris Agreement [98], stricter emissions caps and escalating carbon prices intensified carbon stock spillovers, particularly within carbon-intensive industries. The Market Stability Reserve (MSR), introduced in 2019, aimed to restore the balance between supply and demand while enhancing market resilience [99]. The MSR influences carbon prices by restricting the supply of carbon allowances, thereby incentivizing firms to adopt green technological innovations and reduce their dependence on carbon permits and carbon-intensive energy sources. This dynamic has reshaped the association between the equity and the carbon market returns [1].

Furthermore, Fig 2 also shows that overall volatility shock spillovers between EU-ETS and EU emerging stock markets increases during 2019 and 2022, but this shock spillover effect is more intensified at higher quantiles (bullish volatility period) as compared with the moderate and bearish quantiles. One of the justification is that COVID-19 pandemic temporarily suppressed carbon prices due to reduced industrial activity [78], but subsequent recovery efforts in 2021–2022, coupled with Green Deal objectives, drove increased price volatility. The energy market interruptions precipitated by the 2022 Russia-Ukraine conflict engendered heightened volatility Yousaf et al. [100]. The inclusion of maritime transport emissions within the EU-ETS in 2024 [101] raised compliance costs, may lead to spillovers in shipping and transportation related sectoral stocks. Furthermore, the continuing conflict between Ukraine and Russia exacerbated fluctuations in energy prices, consequently influencing the interrelationship between carbon markets and stock markets. Stricter emissions limit in the new compliance periods of 2024 heightened the demand for allowances, exacerbating price fluctuations [102] and significantly affecting energy-intensive sectors.

The cost structure and profitability expectations of carbon-intensive industries are immediately impacted by changes in EU-ETS prices, which provides theoretical support for the asymmetric quantile domain interactions between the European carbon emission pricing system and stock returns [1]. This suggests that stock markets may react more strongly to carbon-price uncertainty if they are more exposed to carbon-intensive energy supplies and do not internalize sustainable mechanical processes of green energy consumption [3]. Large volatility shocks in carbon pricing raise discount-rate uncertainty, firm profitability, and earnings risk, according to the cost-of-capital and investment-adjustment literature. Therefore, compared to reduced volatility, carbon-intensive businesses may react more strongly and asymmetrically to increased volatility in carbon pricing [3]. This led towards the higher volatility shock spillovers from EU-ETS towards the stocks at bullish volatility states. Additionally, Wen et al. [3] demonstrate that the Chinese financial system is disproportionately and heterogeneously impacted by both increases and decreases in the carbon trading market. Additionally, based on the research on cross-market contagion [43,44], excessive movements in one market cause disproportionate co-movements in others [50] because of liquidity shocks, herding, or common investors (see [45]). As a result, it’s possible that shocks diffused from EU-ETS are tail dependent and that more volatility shock spillovers appear at bullish quantiles [5]. This is because during periods of severe volatility in the carbon market, excessive equity volatility is significantly higher. For instance, Duan et al. [46] also classified that shock spillovers between carbon and financial asset classes are more intensified at varied quantiles. Therefore, this provides the justification of the asymmetrical quantile dependent volatility shock transmission between EU-ETS and EU emerging stock markets.

Furthermore, one of the theoretical justifications that overall volatility shock spillovers between EU-ETS and EU member economies is higher at bullish quantiles as compared with the bearish is that during the bullish volatility states, information arrival is often large and ambiguous. Therefore, noise and informed traders within the heterogeneous-agent frameworks [91] predict that large, novel signals trigger stronger re-pricing as both informed and uninformed agents rapidly update beliefs [45]. During the bullish volatility regime, the speculative and momentum traders amplify price movements, and this has caused the EU-ETS volatility shocks to produce larger contemporaneous re-actions across correlated EU emerging economies equity markets as compared to routine (median) and lower (bearish) volatility periods. The higher volatility shock spillovers across higher volatility regime also comes from the fact that responses of different agent classes are quantile dependent [16]. For instance, in calm volatility regimes (median and bearish quantiles), there may be the possibility that only marginal trading adjusts prices. However, in extreme volatility regime (bullish quantile), there is an occurrence of mass reallocation, escalating the cross-market volatility shock transmission between EU-ETS and stocks.

For portfolio managers, the observed reduction in portfolio diversification advantages, due to the higher volatility shock spillovers between the EU-ETS and European emerging equity markets during bullish volatility periods, underscores the need for proactive hedging strategies. The finding regarding the higher volatility shocks spillovers across bullish volatility regimes implies that traditional, mean-based risk models underestimate systemic exposure to carbon-price shocks. Therefore, traders and policymakers should integrate these interdependencies into regulatory frameworks governing carbon markets to maintain stability amidst fluctuating European emerging equity market volatility. Fund managers and portfolio optimizers adopting a long-term positioning in EU emerging stocks must incorporate the quantile-sensitive forecasting framework for capturing the actual downside risk exposure during extreme market conditions, as EU-ETS volatility shocks are transmitted with more intensity. Similarly, investors must incorporate these asymmetric relationships into their risk assessment models to enhance returns while mitigating potential spillover risks during episodes of extreme equity market turbulence. Additionally, businesses dependent on the EU-ETS for compliance or emissions offsetting should account for the elevated exposure to volatility in European emerging stock markets during bullish trends. This may necessitate updating risk mitigation practices, such as securing carbon allowances during low-stress periods or broadening emissions-reduction initiatives. Furthermore, because spillovers intensify in bullish regimes, policy actions that drive sharp upward movements in EU-ETS prices—such as tightening allowances or new emissions directives—can inadvertently create large, unintended volatility waves in emerging European equity markets. Policymakers should implement more phased, predictable carbon-policy adjustments to reduce cross-market instability and avoid destabilizing these growing but sensitive markets.

### 5.1. The transmission of volatility shocks from the European carbon emission trading system (EU-ETS) towards the European emerging stock markets

Table 2 indicates that a shock in the conditional volatility of the EU-ETS results in significantly higher volatility spillovers to the European emerging stock markets during bullish quantile (τ = 0.95) compared to bearish (τ = 0.05) and moderate (τ = 0.50) quantiles. Specifically, during periods of elevated bullish volatility, a shock in the EU-ETS contributes 95.37% of the error variances to the volatility of other European emerging stock markets, as opposed to 24.55% during bearish conditions and 17.45% during moderate volatility phases. This suggests that the degree of volatility interconnectedness between the EU-ETS and stock markets varies significantly across different volatility quantiles. Furthermore. The EU-ETS also functions as a net shock receiver at lower and median quantiles as seen in(Appendix Figures A1 - A2 in S1 Appendix). Whereas (Appendix Figure A3 in S1 Appendix) shows the net shock transmitter property of EU-ETS across bullish (higher) quantile. Shock transmission between the EU-ETS and established European financial markets has been the main subject of previous research [1,11]. While some research examined symmetrical or linear spillover mechanisms [9,10,29,31,79], these methods ignore tail dependencies that are crucial during periods of calm (moderate quantiles) or market turbulence (extreme bearish or bullish quantiles) [17].

However, previous research by Yadav et al. [60], employing the conventional mean-based connectedness methodology of Diebold and Yilmaz [15], only identified symmetrical transmission of volatility shocks between Chinese stock markets, energy, and carbon emissions. The general results indicated stronger linear interdependencies between the markets over extended periods compared to shorter durations. Conversely, Qiu et al. [42], investigating the reciprocal relationship between carbon and stock markets in Europe using a conventional mean-based shock transmission model, specifically a Time-Varying Parameter Auto-regression (TVP-VAR), observed limited long-run integration between these markets. Furthermore, the flow of shocks from the stock market to the European carbon market becomes more pronounced during periods of financial instability, such as the COVID-19 pandemic. Wu & Jiang [8], employing the TVP-VAR methodology, detected transmission of volatility disturbances between Chinese stock and carbon markets, without taking into account the bearish, bullish and moderate volatility regimes. They posited that these asymmetries stemmed from the greater influence of negative volatility shocks compared to positive ones, rather than the volatility shock propagations across different quantiles of volatility. Meanwhile, Suleman, Rehman, et al. [1] only focused on linear shock transmission between the EU-ETS and European developed economies, revealing only mean-based connectedness based on average returns, rather than volatility shock transmission across quantiles. Additionally, as a contrast to the existing studies, the asymmetric volatility shock transmission from the stock markets of the European Visegrad V4 emerging economies, which exhibit distinct structural and economic characteristics compared to developed stock markets, towards the EU-ETS risk has not been highlighted across varied quantile levels.

Table 2 further reveals that under bearish volatility conditions (τ = 0.05), a shock in the conditional volatility of the EU-ETS leads to increased error variance contributions of 5.27%, 6.32%, and 5.66% to the conditional volatility of the stock markets in Greece, Hungary, and Poland, respectively. Conversely, during moderate volatility conditions (τ = 0.50), an EU-ETS volatility shock results in higher error variance contributions of 5.02%, 4.47%, and 3.67% to the conditional volatility of equity markets in Greece, Hungary, and Poland, respectively. However, the conditional volatility of equity markets in Slovakia and the Czech Republic is minimally impacted by shocks in the EU-ETS volatility during both median and lower volatility periods.

One theoretical explanation for the increased volatility shock transmission from EU-ETS to the equities of EU developing nations at higher quantiles as compared with the bearish and moderate quantiles is that stock market liquidity tends to decline during periods of increasing volatility and uncertainty [47]. Furthermore, because of their relative lack of depth and sophistication, the virtual disappearance of market makers, and their heightened susceptibility to global stock market integration, emerging markets may experience larger liquidity declines during times of financial concern (see Sheikh et al., 2025). Building on this fact, liquidity decreased during periods of increasing volatility, which resulted in greater price implications. Because they are less liquid, emerging markets are more sensitive to periods of increased uncertainty and volatility [40]. Additionally, quantile-based dependence models as proposed by Koenker and Bassett [41] and Koenker and Xiao [103] further elaborated that financial time series exhibit fat tails, asymmetric dependence, and nonlinear shock transmission (see [16,19]). This further supports the quantile domain interactions between EU-ETS and the stock market of EU emerging economies. The financial time series data show a leptokurtic distribution following severe financial system uncertainties like COVID-19 and geopolitical uncertainties like the Russian Ukrainian conflict events, making mean-based models useless for shock transmission. QVAR models find stronger spillovers under high-volatility conditions because reduced liquidity increases the volatility shock transmission effects of EU-ETS shocks on emerging equities during the bullish volatility regime, and this shock transmission effect becomes disproportionately large. As a result, we may conclude that total volatility shock transmission from EU-ETS to the EU emerging stock market is larger at the bullish quantile (τ = 0.95) than at the bearish (τ = 0.05) and moderate quantiles (τ = 0.50).

According to Tabash et al. [16], financial asset classes experience greater shocks at higher quantiles when compared to the bearish and bullish during times of economic uncertainty. Sheikh et al. [50], however, point out that overall shock transmission amongst financial asset classes varies between periods of bullish and bearish markets. Furthermore, the theoretical method presented by Ren et al. [51] incorporates the prominent theoretical reasoning on the volatility shock transmission from EU-ETS towards the EU developing stock markets. For example, there are two primary reasons why merging economies offer a perfect setting for analyzing the effects of volatility in carbon prices. For example, carbon price volatility is a major but little-studied risk since emerging economies depend on carbon trading to satisfy emission objectives. Second, the evolving stock market, which is marked by a high degree of information asymmetry [23,24,45,46] offers a useful framework for examining the asymmetrical impact of carbon pricing uncertainty on stock volatility. This furthermore validates the higher EU-ETS volatility shock transmission towards the EU emerging stocks at bullish quantiles.

These findings about the unequal distribution of EU-ETS volatility shocks affecting the volatility of developing European stock markets have a number of practical ramifications for traders, investors, and shareholders. ***Firstly***, regulators and policy makers have to acknowledge that the EU-ETS plays a crucial role in spreading volatility shocks, particularly in times of positive volatility regimes. This emphasizes the necessity of using quantile-based hedging strategies in conjunction with measures meant to stabilize the EU-ETS during periods of increased market volatility, since its instability may spread to emerging equities markets. Investors in Hungary, Poland, and Greece have the chance to create quantile-specific investing strategies due to the different effects of volatility shocks across bearish and bullish quantiles. The EU emerging stocks of Greece, Hungary, and Poland are most susceptible to shocks to the EU-ETS conditional volatility. This is pertaining to the dynamic beta of these markets in relation to EU-ETS volatility increases in upper-tail regimes. Consequently, the EU-ETS is more vulnerable to changes in carbon-intensive industries including energy, manufacturing, utilities, and industrial sectors. These markets more fully absorb uncertainty generated by carbon prices due to increased absorption of EU-ETS shocks under bullish regimes. Volatility spikes that occur from EU-ETS tightening (lower emission permits) will spread farther into these developing markets, intensify volatility clustering in carbon-sensitive industries, and enhance the endurance of transmitted shocks within national equity markets. Therefore, during times of active EU decarbonization strategy, regulators and policymakers in these nations should anticipate greater procyclical volatility spillovers. This suggests that when EU-ETS volatility starts to shift into high-quantile states, portfolio managers need to rebalance more forcefully since the effective systematic risk contribution upsurges drastically. For instance, investors might strategically direct their resources to less impacted economies like Slovakia and the Czech Republic during bearish, bullish and moderate volatility stages.

***Secondly***, Greece, Hungary, and Poland may be appropriate for short-term or speculative trading methods during the bearish or moderate volatility periods because of their increased susceptibility to EU-ETS volatility. On the other hand, the EU emerging economies’ stocks like Slovakia and the Czech Republic receive the lowest EU-ETS volatility shocks across all quantiles and thereby can provide safe havens for longer-term investments. The quantile domain volatility shock spillovers between EU-ETS and these developing equities become tail-dependent and regime-contingent rather than steady because spillover intensity rises in high-quantile nations. In terms of practical implications, this means that when carbon risk is escalating, the benefits of diversification disappear, covariance structures become state-contingent, and the hedging efficacy of cross-asset portfolios collapses in bullish EU-ETS volatility regimes. Therefore, rather than relying upon the mean-based connectivity models, portfolio managers need to use bearish, bullish and moderate-contingent covariance matrices and QVAR-driven dynamic hedging strategies for portfolio optimization.

Table 2 also shows that a shock in the conditional volatility of EU-ETS lead towards the heightened contribution of volatility shocks of 19.37%, 18.9%, 19.49% and 19.76% towards the conditional volatility of the European emerging equity markets of Czech Republic, Greece, Hungary and Poland, respectively at the higher quantile (τ=0.95). However, the conditional volatility of the Slovakia received the least volatility shock spillovers from EU-ETS during the bullish volatility period (τ=0.95). ***Thirdly***, Slovakia’s stock market is positioned as a strategic choice for hedging due to the reduced EU-ETS spillover effects during times of bullish volatility. The stability of Slovakian markets can be used by investors and fund managers to counteract the higher risks connected to more volatile EU-ETS market. These findings further highlight how crucial exact timing is to speculative trading. Speculators may predict high-quantile spillover effects in emerging equities markets by closely monitoring EU-ETS volatility patterns. This enables them to maximize profits by optimizing entry and exit locations.

***Fourthly***, Table 2 also shows that Hungary, Poland and Slovakia transmitted higher contributions of 19.3%, 18.39% and 18% of conditional volatility shocks towards the conditional volatility in the EU-ETS at the higher quantile (bullish volatility conditions) as compared with Greece (15.43%) and Czech Republic (15.66%), respectively. The disparities in shock transmission across different quantiles underscore the necessity of incorporating quantile-specific volatility forecasts into emissions allowance pricing models such as Poland and Hungry stock markets transmitted higher error variances towards the EU-ETS volatility at median and lower quantiles, whereas Slovakia equity market remain the least volatility shock transmitter. On the contrary, at the higher quantile, Hungary, Poland and Slovakia remain the largest volatility shock transmitters towards the EU-ETS. Such integration would enable the development of more precise pricing mechanisms for the EU-ETS that accurately capture the risk levels associated with European emerging economies’ stock market bearish, bullish and moderate volatility conditions. Furthermore, under conditions of heightened market volatility (higher quantiles), it is imperative for policymakers and regulatory bodies in Slovakia, Hungary, and Poland to allocate increased resources toward mitigating market instability. This could include strengthening regulatory frameworks, improving the transparency of financial disclosures, and implementing tailored interventions aimed at stabilizing the EU-ETS.

***Fifthly***, according to Table 2, a shock in the conditional volatility of Czech Republic, Greece, Hungary and Poland causes highest contributions of 5.04%, 5.44%, 6.57% and 5.56%, respectively of error variances in forecasting the 20-days ahead conditional volatility in the EU-ETS at lower quantile (τ=0.05). Whereas, at the median quantile (τ=0.50), a shock in these economies causes higher contributions of 4.61%, 3.91%, 4.93% and 5.56% of shocks towards the EU-ETS conditional volatility, respectively Under bearish and moderate volatility conditions, disturbances originating from Hungary and Poland equity market volatility exert a disproportionately larger impact on EU-ETS volatility. Therefore, for investors in the EU-ETS, it is essential that investment approaches should prioritize stress-testing at lower and median quantiles, representing extreme downside and moderate risks, given that shocks originating from Hungary and Poland exert a significantly greater influence on EU-ETS volatility in these scenarios. Conversely, during periods of lower or median volatility, shocks to Slovakia’s conditional volatility have the smallest transmission effect on EU-ETS conditional volatility. This suggests that Slovakia may serve as a relatively stable reference market during volatile periods due to its minimal influence on EU-ETS volatility. To enhance regional stability, policymakers could explore aligning Slovakia’s volatility controls and management system with those of other Central and Eastern European emerging stock markets. Additionally, the interconnectedness of the Czech Republic, Greece, Hungary and Poland with the EU-ETS at lower and median quantiles underscores the importance of a harmonized approach to financial regulation and environmental policy. Strengthened cooperation through unified regulations and the exchange of market insights may help alleviate systemic volatility contagion between carbon and stock market, particularly under median and bearish volatility scenarios.

In contrast to these results, Zhang & Xu (2023) pointed out that while symmetrical generalized VAR-based connectedness approaches do not take into account nonlinear dynamics, such as asymmetry and directional dependencies, symmetrical Vector Auto-Regression (VAR) models do capture volatility spillovers across carbon and stock markets. Using mean-based frameworks like VAR, research conducted in China such as Bai et al. [104] and Li et al. [105] examined linear equity return responses to price variations in the emission trading system. In a similar vein, symmetrical spillovers between oil and stocks in carbon-intensive and non-carbon-intensive businesses were examined by Zhang and Xu [14]. However, due to the non-normalcy, fat tails, and skewness of financial time series, quantile-based approaches are more appropriate for capturing asymmetrical shock transmissions between EU-ETS and European emerging markets during extreme events like financial crises or sudden changes in carbon policy [18,24]

#### 5.1.1. The time-varying volatility shock transmission from EU-ETS towards the European emerging stock markets’ conditional volatility.

**Fig 4:**
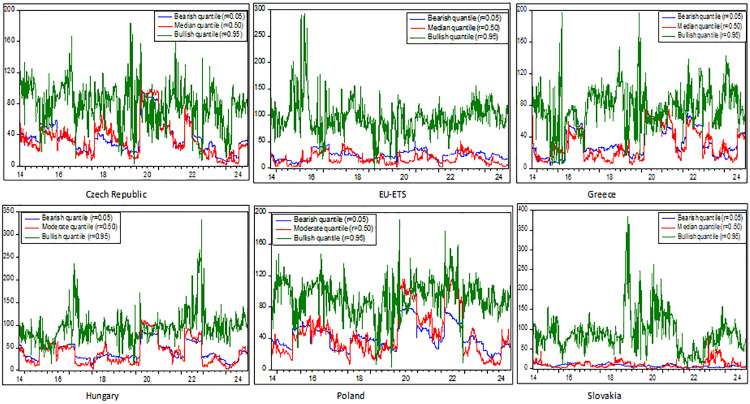
The transmission of conditional volatility shocks from a variable “” towards all other variables “𝐣"at varied quantile levels, i.e., bearish, bullish and moderate.

**Fig 5:**
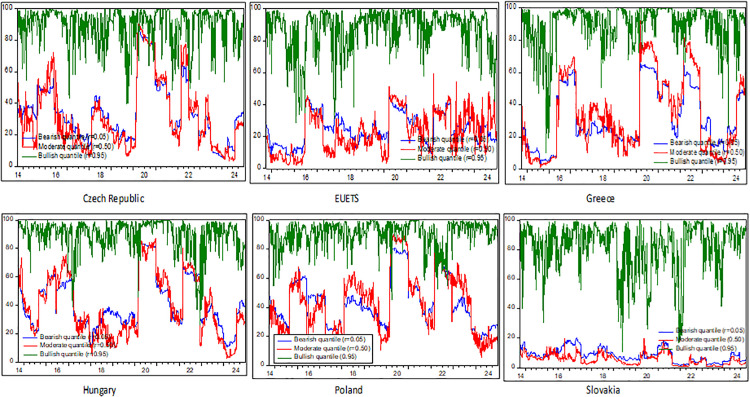
The reception of conditional volatility shocks by a variable “i” FROM all other variables “𝐣" at varied quantile levels, i.e., bearish, bullish and moderate.

**Fig 6:**
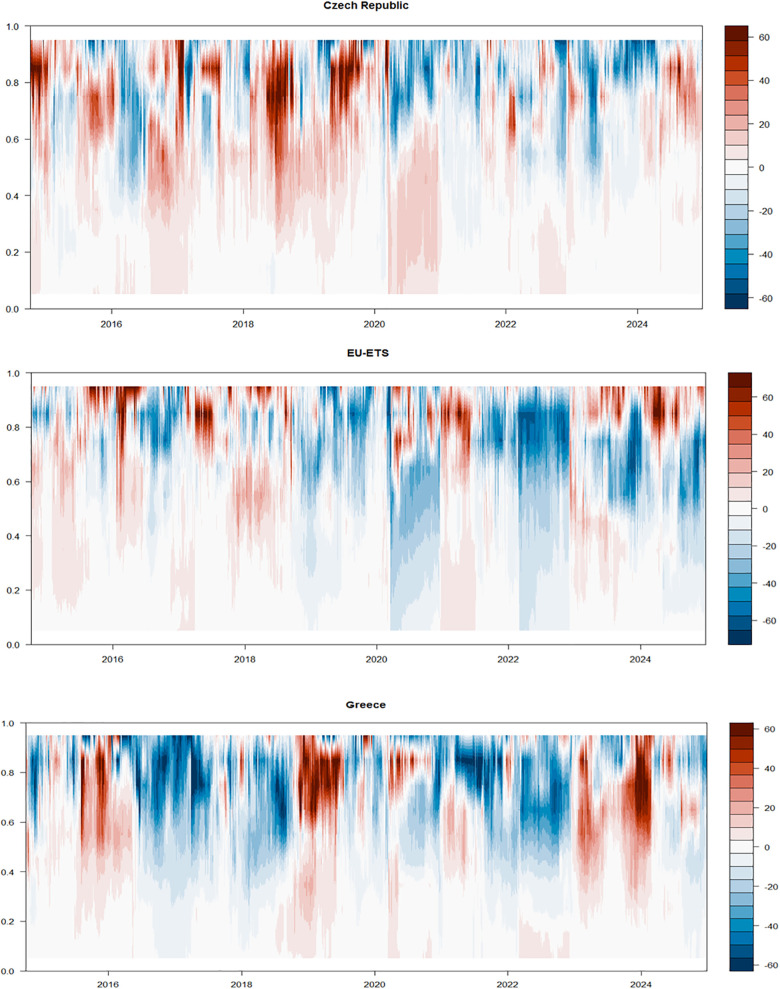
The graphical representation of the heat map for the NET (TO-FROM) volatility shock spillovers between EU-ETS and Emerging economies’ stock markets across quantiles.

**Fig 7:**
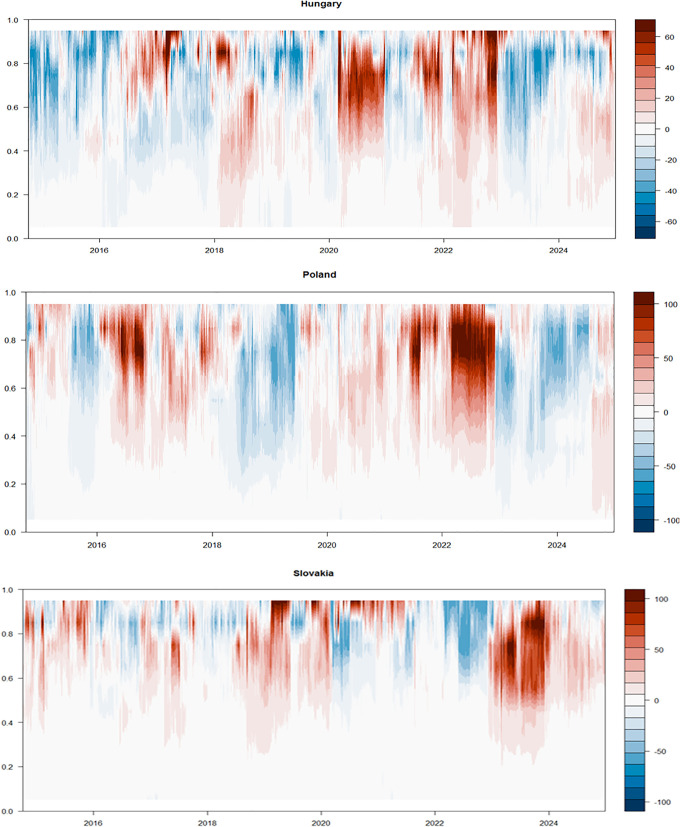
The graphical representation of the heat map for the NET (TO-FROM) volatility shock spillovers between EU-ETS and Emerging economies’ stock markets across quantiles extended.

Fig 4 depicts the dynamic propagation of total forecast error variance stemming from disturbances in the conditional volatility of the EU-ETS to the volatility of stock markets in developing European economies. Notable increases in the transmission of EU-ETS volatility shocks are observed during the periods of 2014–2016, 2018, and the COVID-19 regime, with a pronounced surge evident in 2022, 2023, and 2024. Furthermore, Fig 5 also shows that the EU-ETS received higher volatility shocks from all other EU emerging economies’ stock markets during these specific time frames. Similarly, Figs 6 and 7 together demonstrate that the EU-ETS transmits heightened volatility shock spillovers during 2014–2016, 2018, and the COVID-19 era at higher quantile. Additionally, the heat map depicting net volatility shock spillovers from the EU-ETS reveals a significant increase in the net shocks transmitted during 2023 and 2024 at higher quantile as compared with the lower and median quantiles (see Figs 6 and 7 together).

The transmission of volatility shocks from the EU-ETS between 2014 and 2016 was partially attributable to an excess of available carbon permits [106], a consequence of decreased economic activity following the European sovereign debt crisis, 2008 financial crisis and the expansion of renewable energy sources. The establishment of the Market Stability Reserve (MSR) in 2015 introduced greater ambiguity regarding future carbon pricing, impacting supply mechanisms and further contributing to EU-ETS volatility [107]. As market participants adjusted their strategies, fluctuations in carbon prices were transmitted to stock markets [1]. The Eurozone debt crisis and slow economic recovery amplified stock market sensitivity to carbon price fluctuations, while the 2015 Paris Agreement heightened investor focus on environmental policies, increasing volatility [18]. Volatility shocks during 2014–2016 were further driven by Phase III reforms (2013–2020), which tightened emission caps and reduced allowances, raising carbon prices. This surge created uncertainty for energy-intensive industries, transmitting volatility to the EU emerging economies’ stock prices. For instance, European carbon allowances (EUA) prices rose from around €7/tonne in early 2018 to over €20/tonne by year-end [108]. Additionally, U.S.-China trade tensions in 2018 amplified global financial uncertainty [21], indirectly affecting European market volatility and exacerbating stock price fluctuations in energy and industrial sectors.

The amplified volatility transfers from the EU-ETS to the stock market volatility of developing European economies between 2019 and 2020 can be explained by the sudden policy adjustments triggered by the COVID-19 crisis. During this period, greenhouse gas emissions from stationary installations covered by the EU ETS experienced a remarkable 11.4% drop, falling from 1,530 million tons of CO2 equivalent in 2019–1,355 million tons of CO2 equivalent in 2020, the largest decrease observed since the system’s establishment in 2005. These shifts included government stimulus packages, lockdowns, and trade disruptions, which directly impacted carbon-regulated industries. As a result, these sectors affected the demand for carbon allowances, contributing to greater price fluctuations within the EU-ETS. The situation was further exacerbated in 2022 and 2023 due to energy crises, modifications in environmental regulations, and geopolitical conflicts, such as the Russia-Ukraine war [75]. These external shocks contributed to the rise in EU-ETS volatility, which subsequently spread more broadly to stock markets, especially in emerging European markets.

Table 3 displays the results of the optimal portfolio allocation method, employing the DCC-GARCH model with a *t* copula, demonstrating a considerably greater hedging effectiveness (HE) than the hedge ratio approach. This implies that mitigating long-term volatility within the EU-ETS is more efficiently accomplished by creating optimized portfolio combinations that include at least one of the emerging European stock markets. Consequently, the results presented in Table 3, derived from the optimal portfolio weight selection strategy (OPWSS) developed by Kroner and Ng (1998), demonstrate that establishing such portfolio pairings with emerging European stock markets yields hedging effectiveness (HE) between 64% and 85%, significantly diminishing long-term investment risk related to persistent EU-ETS volatility. Businesses in European emerging economies that are subject to carbon pricing schemes, particularly those that deal in EU-ETS permits, should use the OPWSS instead of the HR method to reduce EU-ETS investment risk as much as possible. Financial planning and operational stability can be further improved by integrating EU emerging stock markets into EU-ETS hedging schemes. Because it dramatically lowers long-term EU-ETS investment risk and offers hedging efficacy ranging from 64% to 85% when the portfolio pairs with one of the EU emerging stock markets, the OPWSS is a strong choice for institutional and retail investors looking to protect their carbon market investments.

**Table 3:** Hedge ratio strategy and optimal portfolio weight selection strategy.

Hedge ratio strategy	Mean	Std.Dev.	5%	95%	Hedging Effectiveness (HE)	p-value	Optimal portfolio weights	Mean	Std.Dev.	5%	95%	Hedging Effectiveness (HE)	p-value
EU-ETS/Czech Republic	0.34	0.16	0.13	0.61	2.0%	0.0100	EU-ETS/Czech Republic	0.12	0.12	0.02	0.39	85.0%	0.0080
EU-ETS/Hungary	0.19	0.1	0.06	0.35	2.0%	0.0000	EU-ETS/Hungary	0.18	0.11	0.05	0.37	79.0%	0.0000
EU-ETS/Poland	0.3	0.11	0.14	0.5	2.0%	0.0000	EU-ETS/Poland	0.17	0.11	0.04	0.37	80.0%	0.0000
EU-ETS/Slovakia	0.06	0.06	−0.04	0.18	0.0%	0.9600	EU-ETS/Slovakia	0.16	0.11	0.04	0.39	89.0%	0.0000
EU-ETS/Greece	0.17	0.08	0.04	0.31	1.0%	0.0000	EU-ETS/Greece	0.34	0.22	0.09	0.84	64.0%	0.0000
Czech Republic/EU-ETS	0.05	0.02	0.02	0.1	5.0%	0.7100	Czech Republic/EU-ETS	0.88	0.12	0.61	0.98	6.0%	0.0000
Hungary/EU-ETS	0.04	0.02	0.01	0.08	5.0%	0.7100	Hungary/EU-ETS	0.82	0.11	0.63	0.95	14.0%	0.0000
Poland/EU-ETS	0.07	0.03	0.03	0.12	3.0%	0.7100	Poland/EU-ETS	0.83	0.11	0.63	0.96	12.0%	0.0000
Slovakia/EU-ETS	0.01	0.01	−0.01	0.03	0.0%	0.7100	Slovakia/EU-ETS	0.84	0.11	0.61	0.96	12.0%	0.0000
Greece/EU-ETS	0.09	0.07	0.03	0.2	3.0%	0.7100	Greece/EU-ETS	0.66	0.22	0.16	0.91	54.0%	0.0000

Note: This table compares the maximum hedging effectiveness for EU-ETS volatility between the hedge ratio strategy ($\beta_{SI.EUETS,t}=\frac{h_{SI.EUETS,t}}{h_{SI,t}}$) in Eq. 16 and the optimal portfolio weight selection approach ($w_{SI.ETS,t\;}=\;\frac{h_{EUETS,t}-h_{SI.EUETS,t}}{h_{SI,t}-\;{\text{2}h}_{SI.EUETS,t}+h_{EUETS}}$ in Eq. 19) based on the DCC-GARCH-t copula method. The hedge ratio strategy determines whether short-positioning in one of the EU emerging stock markets provides higher hedging effectiveness against long-term EU-ETS volatility, and vice versa. Meanwhile, the optimal portfolio weight selection strategy assesses whether forming optimal portfolio pairs with EU emerging stock markets maximizes risk reduction against EU-ETS volatility.

The ideal portfolio weights for EU-ETS risk hedging fall between $0.12 and $0.32, as Table 3 demonstrates. This suggests that in a $1 portfolio consisting of EU-ETS and the emerging stock markets of Slovakia and the Czech Republic, respectively, $0.88 and $0.84 should be allocated to the emerging stock returns in order to maximize risk reduction for EU-ETS. The main reason for this is that optimizing portfolio weights with Slovakia’s and the Czech Republic’s developing stock markets yields better hedging effectiveness (HE) of 89% and 85%, respectively, necessitating a larger allocation to these markets in order to hedge EU-ETS volatility. Furthermore, Table 3 shows that for maximal HE against EU-ETS risk, larger amounts of $0.82, $0.83, and $0.66 should be invested in the stock markets of Hungary, Poland, and Greece, respectively, in a $1 portfolio of EU-ETS and developing stock markets. The Greek market is the most economically beneficial option among these developing European equity markets for reducing long-term EU-ETS risk by building an optimal portfolio that combines Greek stocks with EU-ETS instruments. As a result, the Greek stock market is the most cost-effective option for protecting against ongoing EU-ETS risk. For investors who prioritize cost-effective techniques, a portfolio allocation of 0.66 units in Greek stocks presents an attractive alternative since it offers significant risk reduction.

Therefore, the findings highlight that optimal portfolio formulation with EU emerging stock markets provides markedly superior hedging effectiveness for the EU-ETS. Furthermore, the Table 3 also shows that the higher HE is ranging in between 6% and 64% for the EU stock markets can be achieved by hedging the stock market risk through formulating portfolio pairs with the EU-ETS. In contrast, the hedge ratio (HR) method, as formulated by Kroner and Sultan (1993) and presented in Table 3, proves considerably less effective at hedging, achieving only between 0% and 2% risk reduction for long-term positions within the EU-ETS by taking short positions in European stock markets. Similarly, using the HR strategy to hedge long-term risk in emerging European stock markets via short-term EU-ETS positions yields similarly poor results, with effectiveness ranging from a mere 0% to 5%.

### 5.2. Robustness analysis of quantile domain shock spillovers

Appendix Figure A4 in S1 Appendix graphically illustrates the robustness of quantile domain shock spillovers between EU-ETS and EU member economies’ stock markets across different H-step ahead forecasting horizons (20 and 25) and multiple quantiles, i.e., bearish (τ=0.05), bullish (τ=0.05) and median (τ=0.50). Overall findings suggested that overall total connectedness indices (TCI) between the conditional volatility of EU-ETS and EU emerging economies’ stock markets are consistent and reliable as exhibiting similar tendency and spillover pattern across multiple H-step ahead forecasting horizon (H = 20 and H = 25). This consistent of spillover of volatility shocks across different prediction widows (20 and 25) validates the stability of the underlying asymmetrical carbon-stock shock transmission mechanism. The total connectedness indices (TCI) between the conditional volatility of EU-ETS and stock markets is represented as the aggregated value of the forecast error variances within the entire QVAR system and thereby represented the dynamic volatility diffusion and information transmission across interconnected carbon trading and EU emerging economies’ financial systems. The TCI are expected to evolve smoothly over time rather than being highly sensitive to marginal changes in the H-step ahead forecasting horizon. Therefore, the consistency of quantile domain asymmetrical shock spillovers across different forecasting horizons, i.e., H = 20 and H = 25 characterizes the stability of volatility shock transmission, reception and endured interdependencies rather than highly susceptible to different H-step ahead forecasting horizons. Regardless of the dependence of quantile domain EU-ETS-stock market shock spillovers across H-step ahead forecasting horizons, the similarity of spillover patterns suggests that the system has reached a convergence region due to the reduction of sensitivity to arbitrary horizon selection and mitigating concerns related to model instability. The consistency of shock spillovers across different forecasting horizons also causes the elimination of estimation bias, overfitting, enhances the reliability, robustness, and external validity of the empirical findings. This has confirmed that the estimated shock spillover mechanism capture genuine economic interrelationship between EU-ETS and EU emerging economies’ stock market risk rather than artifacts of model specification.

According to the graphical representation of the Appendix Figures (A5-A7) in S1 Appendix, the asymmetrical quantile domain shock spillover pattern between conditional volatility of EU-ETS and equity market is consistent across different rolling windows (150 and 200), across bearish, median, and bullish market regimes, respectively. The validation of the consistent shock spillovers across different rolling windows during bearish, median and bullish quantiles ensures the stability and reliability of the total connectedness indices (TCI) estimated through QVAR with “Extended Joint” connectivity approach. The selection of rolling windows for the overall shock spillovers between EU-ETS and EU emerging economies’ stock market conditional volatility ensures how the model captures time-varying relationships and structural changes within the variables. The fact that the total connectedness indices exhibit similar patterns across both rolling windows implies that the results are not driven by window-specific estimation bias, small-sample distortions and the underlying parameter estimates are stable over time. Furthermore, the consistency of quantile domain shock spillovers across different rolling windows ensures that the QVAR model based dynamic spillover structure has sufficient persistence such that moderate changes in the estimation window do not alter the inferred transmission mechanism. The consistency of quantile domain shock spillovers across bearish median and bullish quantiles enhances confidence in the internal validity of the model, as it demonstrates that the findings are robust to alternative specifications of the rolling estimation procedure and the connectedness structure is not regime-dependent in a fragile or unstable way. This also led towards the reduction in concerns about structural breaks or regime-specific parameter instability. This also implies that the QVAR model captures a coherent data-generating process across varying market conditions and the estimated total shock spillovers are reliable, not artifacts of arbitrary methodological choices, and possess strong external validity for inference and policy interpretation.

## 6. Conclusion with Policy Guidelines, limitations and future research directions

This research constitutes the initial investigation into quantile-specific “Extended Joint” connectedness between the conditional volatility of the European Union Carbon Emission Trading System (EU-ETS) and MSCI emerging equity markets within the European Union (specifically, those of the Czech Republic, Hungary, Poland, Slovakia, and Greece). Utilizing the Quantile-based Vector Autoregression (QVAR) methodology within the “Extended Joint” connectedness framework developed by Cunado et al. [38], the study analyzes extreme volatility spillover dynamics across median, bearish, and bullish quantiles. The combination of the QVAR-based connectedness approach of Ando et al. [40] with the “Extended Joint” framework of Balcilar et al. [41] offers improved normalization, greater resilience to outliers, and enhanced interpretability relative to traditional QVAR connectedness methods [89]. Furthermore, this study compares a hedge ratio strategy with an optimal portfolio weight selection strategy, employing a DCC-GARCH model with a t copula, to assess the efficacy of hedging long-term EU-ETS risk through investments in emerging EU equity markets.

The results reveal that the impact of volatility shocks transmitted between the EU-ETS and emerging European financial markets is greater during periods of high volatility (upper quantiles) than during periods of moderate or low volatility (median and lower quantiles). For portfolio managers, the observed reduction in portfolio diversification advantages, due to the higher volatility shock spillovers between the EU-ETS and European emerging equity markets during bullish volatility periods, underscores the need for proactive quantile domain hedging strategies. The finding regarding the higher volatility shocks spillovers across bullish volatility regimes implies that traditional, mean-based risk models underestimate systemic exposure to carbon-price shocks. Therefore, traders and policymakers should integrate these interdependencies into regulatory frameworks governing carbon markets to maintain stability amidst fluctuating European emerging equity market volatility. Fund managers and portfolio optimizers adopting a long-term positioning in EU emerging stocks must incorporate the quantile-sensitive forecasting framework for capturing the actual downside risk exposure during extreme market conditions, as EU-ETS volatility shocks are transmitted with more intensity. Similarly, investors must incorporate these asymmetric relationships into their risk assessment models to enhance returns while mitigating potential spillover risks during episodes of extreme equity market turbulence. Additionally, businesses dependent on the EU-ETS for compliance or emissions offsetting should account for the elevated exposure to volatility in European emerging stock markets during bullish trends. This may necessitate updating risk mitigation practices, such as securing carbon allowances during low-stress periods or broadening emissions-reduction initiatives. Furthermore, because spillovers intensify in bullish regimes, policy actions that drive sharp upward movements in EU-ETS prices—such as tightening allowances or new emissions directives—can inadvertently create large, unintended volatility waves in emerging European equity markets.

Furthermore, disturbances in the conditional volatility of the EU-ETS account for the largest proportion of forecast error variance in the conditional volatility of the Hungarian, Polish, and Greek stock markets at low and moderate volatility levels. Conversely, the Czech and Slovakian equity markets demonstrate minimal sensitivity to EU-ETS volatility shocks at these same levels. Furthermore, overall QVAR based findings also showed that Poland, Hungary, Greece and Czech Republic also receive the highest volatility shock spillovers across bullish quantile as compared with the median and bearish quantile, whereas Slovakia’s stock market conditional volatility receive the least shock spillovers from EU-ETS. This means that Slovakian equity market’s conditional volatility receives the lowest volatility shocks from EU-ETS during all the quantiles. Investors in Hungary, Poland, and Greece have the chance to create quantile-specific investing strategies due to the different effects of volatility shocks across bearish and bullish quantiles. The EU emerging stocks of Greece, Hungary, and Poland are most susceptible to shocks to the EU-ETS conditional volatility. This is pertaining to the dynamic beta of these markets in relation to EU-ETS volatility increases in upper-tail regimes. Therefore, during times of active EU decarbonization strategy, regulators and policymakers in these nations should anticipate greater procyclical volatility spillovers. This suggests that when EU-ETS volatility starts to shift into high-quantile states, portfolio managers need to rebalance more forcefully since the effective systematic risk contribution upsurges drastically. For instance, investors might strategically direct their resources to less impacted economies like Slovakia and the Czech Republic during bearish, bullish and moderate volatility stages. Greece, Hungary, and Poland may be appropriate for short-term or speculative trading methods during the bearish or moderate volatility periods because of their increased susceptibility to EU-ETS volatility. On the other hand, the EU emerging economies’ stocks like Slovakia and the Czech Republic receive the lowest EU-ETS volatility shocks across all quantiles and thereby can provide safe havens for longer-term investments.

Conversely, at higher quantiles (τ = 0.95), EU-ETS conditional volatility shocks result in the greatest volatility spillover effects on the equity markets of the Czech Republic, Greece, Hungary, and Poland. Whereas Slovakia’s equity market remains the least influenced by the transmission of EU-ETS volatility shocks. Therefore, during bullish conditions (τ = 0.95), policy makers should implement stronger oversight and measures like circuit breakers, stimulus packages, and volatility controls in Hungary, Poland, Greece, and the Czech Republic to stabilize stock markets, prevent systemic disruptions, and boost investor confidence. Under conditions of high volatility in the EU-ETS market (τ=0.95), portfolio managers are advised to diversify their stock portfolios by allocating investments to the Slovak financial markets, as this exhibit reduced susceptibility to shocks associated with the EU-ETS. In terms of practical implications, the higher volatility shock transmission between EU-ETS and EU emerging economies’ stock volatility means that when carbon risk is escalating, the benefits of diversification disappear, covariance structures become state-contingent, and the hedging efficacy of cross-asset portfolios collapses in bullish EU-ETS volatility regimes. Therefore, rather than relying upon the mean-based connectivity models, portfolio managers need to use bearish, bullish and moderate-contingent covariance matrices and QVAR-driven dynamic hedging strategies for portfolio optimization. Slovakia’s stock market is positioned as a strategic choice for hedging due to the reduced EU-ETS spillover effects during times of bullish volatility. The stability of Slovakian markets can be used by investors and fund managers to counteract the higher risks connected to more volatile EU-ETS market. These findings further highlight how crucial exact timing is to speculative trading. Speculators may predict high-quantile spillover effects in emerging equities markets by closely monitoring EU-ETS volatility patterns. This enables them to maximize profits by optimizing entry and exit locations.

The findings regarding the quantile domain volatility shock transmission from EU emerging economies’ stock markets towards the EU-ETS volatility indicate that conditional volatility in the stock markets of Greece, Hungary, Poland, and the Czech Republic contributes significantly to volatility shocks in the EU-ETS under bearish and moderate market volatility conditions. Conversely, the Slovakian stock market transmits lowest volatility shocks during these periods. However, under bullish market conditions, the equity markets in Hungary, Poland, and Slovakia exhibit higher transmission of volatility shocks to the EU-ETS, while the Czech Republic and Greece contribute the least volatility shocks to the EU-ETS. Therefore, during bullish conditions, regulators should implement temporary liquidity support for carbon market participants, such as increasing allowance auctions or introducing reserve allowances. Additionally, strengthening macro prudential regulation in Hungary, Poland, and Slovakia through risk monitoring and stress testing can help prevent volatility shocks in the EU-ETS during bullish volatility conditions.

The findings reveal that the optimized portfolio allocation methodology, employing the DCC-GARCH framework with a t copula, yields markedly greater risk mitigation efficacy than the conventional hedge ratio technique. This demonstrates that hedging for the EU-ETS is more effectively achieved by constructing optimal portfolio pairs with one of the EU emerging stock markets. Policymakers and regulators should focus on fostering deeper integration of EU emerging stock markets to enhance hedging effectiveness against EU-ETS volatility and promote investments in the Czech Republic, Slovakia, Hungary, Poland, and Greece. Notably, the equity markets of the Czech Republic and Slovakia should be prioritized for their superior hedging effectiveness of 85% and 89%, respectively, against EU-ETS volatility. Additionally, the Greek equity market is a cost-effective option for mitigating long-term EU-ETS risks. Policy makers should provide financial incentives or tax benefits to investors incorporating stocks from Greece, the Czech Republic, and Slovakia into their hedging portfolios as this could strengthen financial stability while ensuring robust protection against long-term EU-ETS volatility and regulatory uncertainties in the carbon market. Furthermore, regulators and policy makers should also reduce barriers and improve access to cross-market investments in emerging European stock markets, and this can significantly enhance hedging efficiency and bolster resilience against EU-ETS market shocks.

Future studies should explore whether EU-ETS volatility shocks have a stronger impact on positive volatility in European developed equity markets compared to negative volatility. Positive (good) volatility is linked to stock market rallies, while negative (bad) volatility signals downturns (Tabash et al., 2024). Analyzing these uneven stock market transmission effects could offer crucial information for investors and regulatory bodies in controlling market instability across diverse volatility regimes (both positive and negative) within the established financial framework. Furthermore, one of the limitations of the study is to analyses the volatility shock spillovers across quantiles, without taking into account the heterogeneous responses of shocks to varied frequency wavelengths. Therefore, future studies should take into account the QVAR based “Frequency” connectedness approach of Chanziantoniou et al. [109] and explore the volatility shock spillovers across quantiles for the short-term and long-term.

## Supporting information

S1 Appendix Supplemental figures and table. This appendix includes seven figures (Appendix Figures A1-A7) and one table (Appendix Table A1) [110].
(DOCX)
