# Supplementary material for: The quantile domain volatility shock transmission between carbon emission trading system and European emerging stock markets: Practical implications for portfolio optimization
Source: PLoS One. 2026 Jun 8;21(6):e0349789. doi: 10.1371/journal.pone.0349789 (PMC13245802; doi:10.1371/journal.pone.0349789)
Supplement: S1 Appendix — This appendix includes seven figures (Appendix Figures A1-A7) and one table (Appendix Table A1) [110]. (DOCX) [file pone.0349789.s001.docx]

**Appendix Table A1**

**Table A1:** BDS test of independence, identical distribution, and non-linearity by Brock et al. (1996)

|  | Czech Republic | |  |  |  | EU-ETS |  |  |  |  | Greece |  |  |  |
| --- | --- | --- | --- | --- | --- | --- | --- | --- | --- | --- | --- | --- | --- | --- |
| Dimension | BDS Statistic | Std. Error | z-Statistic | Prob. |  | BDS Statistic | Std. Error | z-Statistic | Prob. |  | BDS Statistic | Std. Error | z-Statistic | Prob. |
| 2 | 0.184*** | 0.002 | 97.958 | 0.000 |  | 0.178*** | 0.002 | 107.308 | 0.000 |  | 0.201*** | 0.002 | 89.497 | 0.000 |
| 3 | 0.312*** | 0.003 | 104.014 | 0.000 |  | 0.300*** | 0.003 | 113.689 | 0.000 |  | 0.342*** | 0.004 | 95.479 | 0.000 |
| 4 | 0.397*** | 0.004 | 111.267 | 0.000 |  | 0.381*** | 0.003 | 121.386 | 0.000 |  | 0.440*** | 0.004 | 102.831 | 0.000 |
| 5 | 0.454*** | 0.004 | 121.780 | 0.000 |  | 0.433*** | 0.003 | 132.586 | 0.000 |  | 0.508*** | 0.004 | 113.470 | 0.000 |
| 6 | 0.490*** | 0.004 | 136.127 | 0.000 |  | 0.466*** | 0.003 | 147.887 | 0.000 |  | 0.554*** | 0.004 | 127.980 | 0.000 |
|  | Hungary |  |  |  |  | Poland |  |  |  |  | Slovakia |  |  |  |
| Dimension | BDS Statistic | Std. Error | z-Statistic | Prob. |  | BDS Statistic | Std. Error | z-Statistic | Prob. |  | BDS Statistic | Std. Error | z-Statistic | Prob. |
| 2 | 0.169*** | 0.002 | 84.906 | 0.000 |  | 0.181*** | 0.002 | 103.161 | 0.000 |  | 0.165*** | 0.002 | 97.715 | 0.000 |
| 3 | 0.283*** | 0.003 | 89.550 | 0.000 |  | 0.307*** | 0.003 | 109.748 | 0.000 |  | 0.274*** | 0.003 | 102.334 | 0.000 |
| 4 | 0.357*** | 0.004 | 94.955 | 0.000 |  | 0.391*** | 0.003 | 117.559 | 0.000 |  | 0.343*** | 0.003 | 107.798 | 0.000 |
| 5 | 0.403*** | 0.004 | 102.808 | 0.000 |  | 0.447*** | 0.003 | 128.895 | 0.000 |  | 0.384*** | 0.003 | 115.971 | 0.000 |
| 6 | 0.429*** | 0.004 | 113.600 | 0.000 |  | 0.482*** | 0.003 | 144.312 | 0.000 |  | 0.407*** | 0.003 | 127.302 | 0.000 |

Note: This Table explains the BDS test of non-linearity. The rejection of null hypothesis of independence and identical distribution of EU-ETS and EU member economies’ stock market conditional volatility series implies the presence of non-linearity. The asterisks sign of *** implies the rejection of null hypothesis due to the p-values less than 1% level of significance.

**Appendix Figure A1**


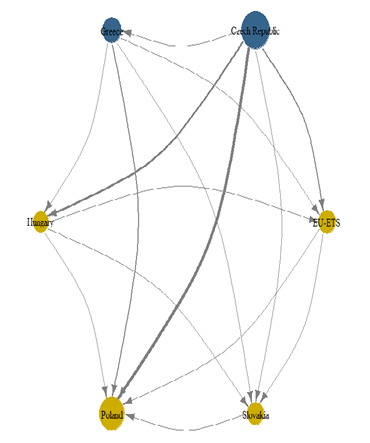


Fig. A1 Network of volatility shocks between EU-ETS and stock markets at lower quantiles

**Appendix Figure A2**


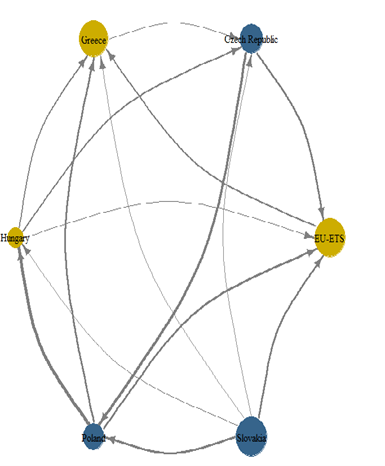


Fig. A2 Network of volatility shocks between EU-ETS and stock markets at median quantiles

**Appendix Figure A3**


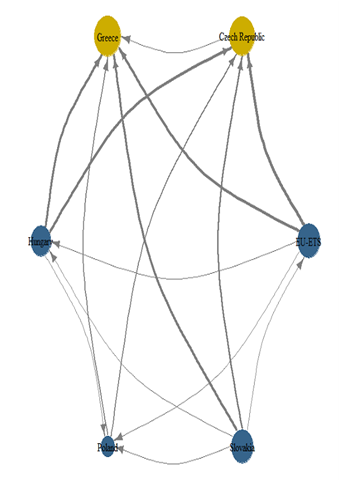


Fig. A3 Network of volatility shocks between EU-ETS and stock markets at bullish quantiles

**Appendix Figure A4**


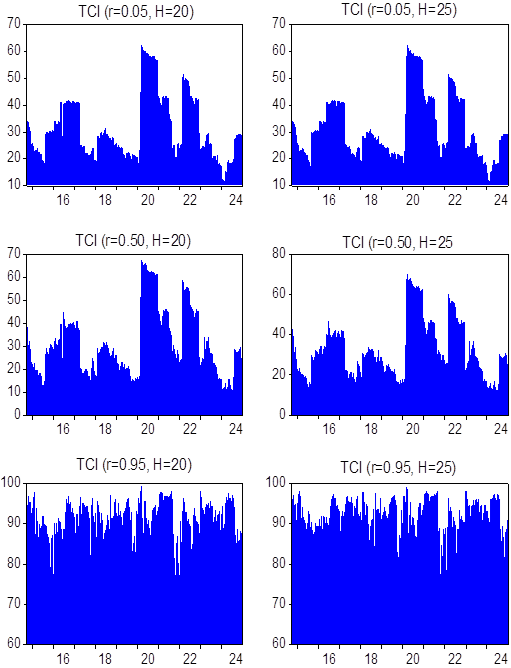


Fig. A4 Quantile domain shock spillovers between EU-ETS and EU emerging economies’ stock market volatility across different H-step ahead forecasting horizons (H=20, 25)

**Appendix Figure A5**


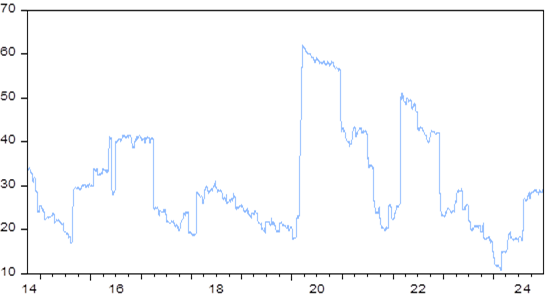


Fig. A5 The quantile domain shock spillovers between EU-ETS and EU emerging economies’ stock market volatility across bearish quantile ($\tau=0.05)$and different rolling windows (150 and 200)

**Appendix Figure A6**


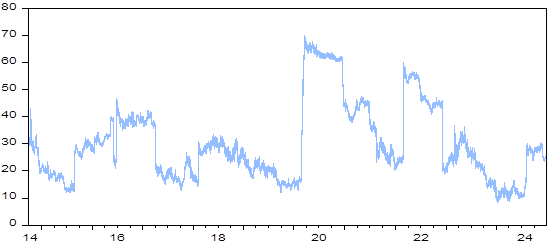


Fig. A6 The quantile domain shock spillovers between EU-ETS and EU emerging economies stock market volatility across median quantile ($\tau=0.50)$ and different rolling windows (150 and 200)

**Appendix Figure A7**


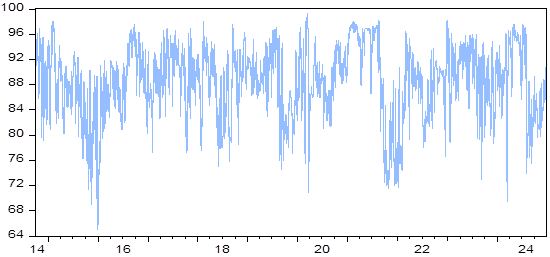


Fig. A7 The quantile domain shock spillovers between EU-ETS and EU emerging economies stock market volatility across bullish quantile ($\tau=0.95)$ and different rolling windows (150 and 200
